# Age-stratified associations between radiotherapy and SPMs for FPHNC: a population-based cohort study

**DOI:** 10.1186/s40779-025-00612-4

**Published:** 2025-05-21

**Authors:** Yuan-Yuan Li, Qiong Liu, Si-Qi Ying, Xiu-Quan Wu, Xiao-Hui Zhang, Xiao-Mei Xie, Bing-Dong Sui, Yan Jin, Yang Jiao, Franklin R. Tay

**Affiliations:** 1 State Key Laboratory of Oral and Maxillofacial Reconstruction and Regeneration, National Clinical Research Center for Oral Diseases, Shaanxi Key Laboratory of Stomatology, Department of Prosthodontics, School of Stomatology, the Fourth Military Medical University, Xi’an, 710032 China; 2 Department of General Dentistry, Xiamen University Affiliated Chenggong Hospital, the 73rd Army Hospital of Chinese PLA, Xiamen, 361001 Fujian China; 3 State Key Laboratory of Oral and Maxillofacial Reconstruction and Regeneration, National Clinical Research Center for Oral Diseases, Shaanxi International Joint Research Center for Oral Diseases, Center for Tissue Engineering, School of Stomatology, the Fourth Military Medical University, Xi’an, 710032 China; 4 Department of Orthopedics, Xijing Hospital, the Fourth Military Medical University, Xi’an, 710032 China; 5 Department of Neurosurgery, Xijing Hospital, the Fourth Military Medical University, Xi’an, 710032 China; 6 State Key Laboratory of Oral and Maxillofacial Reconstruction and Regeneration, National Clinical Research Center for Oral Diseases, Shaanxi Clinical Research Center for Oral Diseases, Department of Orthodontics, School of Stomatology, the Fourth Military Medical University, Xi’an, 710032 China; 7 Department of Stomatology, the Seventh Medical Center of PLA General Hospital, Beijing, 100700 China; 8 Department of Endodontics, the Dental College of Georgia, Augusta University, Augusta, GA 30912-1129 USA

**Keywords:** Population-based cohort study, Head and neck cancer (HNC), Second primary malignancy (SPM), Radiotherapy, Age

## Abstract

**Background:**

Second primary malignancies (SPMs) account for over 30% of total deaths in head and neck cancer (HNC) patients. The increasing use of radiotherapy raises concerns about the elevated risk of radiation-associated SPMs. This study aimed to investigate the age-stratified association between radiotherapy and SPM risk in survivors of non-metastatic primary HNC.

**Methods:**

Using data from the Surveillance, Epidemiology, and End Results program (2004−2015), incidence rate ratios (IRRs) and standardized incidence ratios (SIRs) were evaluated for solid and hematologic SPMs associated with radiotherapy within different age groups. Follow-up for hematologic and solid SPMs began 2 and 5 years, respectively, after the diagnosis of first primary HNC. The IRRs for SPMs were compared between radiotherapy-exposed and unexposed groups using multivariable modified Poisson regression. The SIRs were computed as the ratio of observed cancers in the cohort to expected cases derived from sex-, age-, and calendar year-matched general population incidence rates.

**Results:**

The study included 75,209 2-year survivors, with 73.2% being male and a median age of 60 years. Of these, 58,063 had survived 5 years or more. Radiotherapy was associated with an increased risk of solid SPMs [IRR = 1.16, 95% confidence interval (CI) 1.08−1.24; *P* < 0.001]. The associations varied significantly among young (aged 15−39 years), middle-aged (aged 40 − 64 years), and elderly (aged 65−89 years) patients. Specifically, radiotherapy was associated with an increased risk of solid SPMs in middle-aged patients (IRR = 1.21, 95% CI 1.11−1.32; *P* < 0.001), and a decreased risk of hematologic SPMs in elderly patients (IRR = 0.77, 95% CI 0.60−0.99; *P* = 0.045). Compared with the general population, young patients had an elevated risk of radiotherapy-associated second primary non-Hodgkin lymphoma (SIR = 4.01, 95% CI 1.47−8.74). Middle-aged patients showed the highest SIR for SPMs in the bones/joints (SIR = 7.72, 95% CI 4.32−12.73), while elderly patients had the highest SIR for second primary esophageal malignancies (SIR = 3.87, 95% CI 2.91−5.05). Males were more likely to develop solid SPMs compared to females.

**Conclusions:**

This study reveals an age-stratified association between radiotherapy and the risk of SPMs in HNC patients. These findings highlight the importance of considering patient age when making treatment decisions for HNC and suggest that long-term surveillance is necessary for high-risk groups.

**Supplementary Information:**

The online version contains supplementary material available at 10.1186/s40779-025-00612-4.

## Background

Head and neck cancer (HNC) is the seventh most common malignancy worldwide, with over 870,000 new cases diagnosed and approximately 440,000 deaths reported in 2018 [1]. It encompasses all malignancies affecting the upper aerodigestive tract [2]. Although HNC is typically diagnosed in individuals over the age of 65, there has been a notable rise in incidence among younger populations [3]. Recent epidemiological data revealed a concerning rise in HNC incidence in middle-aged adults (40−64 years) in the United States (US), with rates increasing from 16.7 cases per 100,000 population in 2000 to 17.4 per 100,000 population in 2021 [4].

Radiotherapy is an important adjuvant treatment for most HNCs [5, 6]. Although radiotherapy has greatly improved therapeutic outcomes, there are risks associated with its use. The cell-killing effects of radiotherapy are not limited to cancer cells; they also cause chromosomal abnormalities or mutations in healthy cells [7, 8]. Previous studies reported that radiotherapy is associated with increased risks of short- and long-term adverse outcomes in different types of cancer, including second primary malignancies (SPMs) [9–11]. It has been identified as the leading cause of non-HNC-related deaths, accounting for over 30% of total fatalities. This is 3 times the number of deaths caused by distant metastases [12]. Notwithstanding, few studies have examined the association between radiotherapy and SPM incidence in HNC patients, and the findings have been equivocal [13–16]. The lack of conclusive evidence may stem from limitations such as small sample sizes, short follow-up periods, inadequate consideration of confounding factors such as chemotherapy, or the latency period between radiotherapy and the onset of SPMs [16].

The risk of radiation-related effects is particularly high for younger individuals due to their increased tissue susceptibility and longer life expectancy [16, 17]. Prior studies have shown an increased risk of solid SPMs in this population [16, 17], underscoring the need for age-based treatment decisions and long-term surveillance in HNC. However, current treatment guidelines inadequately address age-specific differences and lack tailored follow-up protocols for SPMs in HNC patients across age groups [18]. This discrepancy between evidence and practice highlights the urgent need to refine therapeutic approaches and surveillance strategies to mitigate age-related risks in HNC survivors.

Cohort studies are essential for investigating the associations between radiation exposure and cancer prognosis [19]. These studies enable detailed data collection and comprehensive evaluation of risk factors. In this context, the present cohort study utilized records from population-based cancer registries to estimate the risk of SPMs associated with radiotherapy in primary HNC, with a particular emphasis on age. Confounding factors such as chemotherapy and latency period were thoroughly accounted for.

## Methods

This study adhered to the Strengthening the Reporting of Observational Studies in Epidemiology (STROBE) checklist [20].

### Data source

This cohort study analyzed data from the Surveillance, Epidemiology, and End Results (SEER) Program (November 2023 update), utilizing research records from 17 registries [21]. The SEER 17 registries database represents approximately 26.5% of the US population. This population-based database includes registries from San Francisco (SF)-Oakland Metropolitan Statistical Area (MSA), Connecticut, Hawaii, Iowa, New Mexico, Seattle (Puget Sound), Utah, Atlanta (Metropolitan), San Jose-Monterey (SJM), Los Angeles (LA), Rural Georgia, California (excluding SF/SJM/LA), Kentucky, Louisiana, New Jersey, and Greater Georgia.

### Cohort definition

The study included individuals diagnosed with first primary head and neck cancer (FPHNC) between January 1, 2004, and December 31, 2015. These individuals were aged 15 − 89 years at the time of diagnosis. The eligibility criteria of HNC were based on the 2023 revision of the International Classification of Diseases for Oncology (ICD-O)−3 site recode (Additional file 1: Table S1). Only malignant cases were considered. Patients without metastatic disease at diagnosis (M0) were included, with the M0 status defined according to the American Joint Committee on Cancer 6 th edition Tumor, Node, and Metastasis (TNM) classifications. Exclusion criteria included: 1) cases identified solely through death certificates or autopsies; 2) cases with metastasis or unknown metastatic stage.

### Exposure and outcome

Radiotherapy was defined as receiving “beam radiation”. In the SEER database, radiotherapy was classified as “yes” or “none/unknown”, with the latter indicating no documented evidence of radiotherapy in the medical records. The outcome of interest was the development of SPM. The latter was defined as a new, metachronous invasive malignancy occurring after the diagnosis of FPHNC, as detailed in previous studies [15–17]. The primary focus was on evaluating both solid and hematologic SPMs. These malignancies were categorized by site (Additional file 1: Table S2).

To differentiate SPMs from multiple reports, relapses, and metastases of the first primary cancer, analyses were limited to non-HNC SPMs [17, 22]. Follow-up for hematologic and solid SPMs began 2 and 5 years, respectively, after the diagnosis of FPHNC. These represent the minimal latency periods for ionizing radiation-induced carcinogenesis [23]. For each patient, the observation period spanned from the date of FPHNC diagnosis until the earliest occurrence of any of the following: diagnosis of a non-HNC SPM, death from any cause, or the end of the study period (December 31, 2021).

### Statistical analyses

#### Data sources and description

**Fig. 1:**
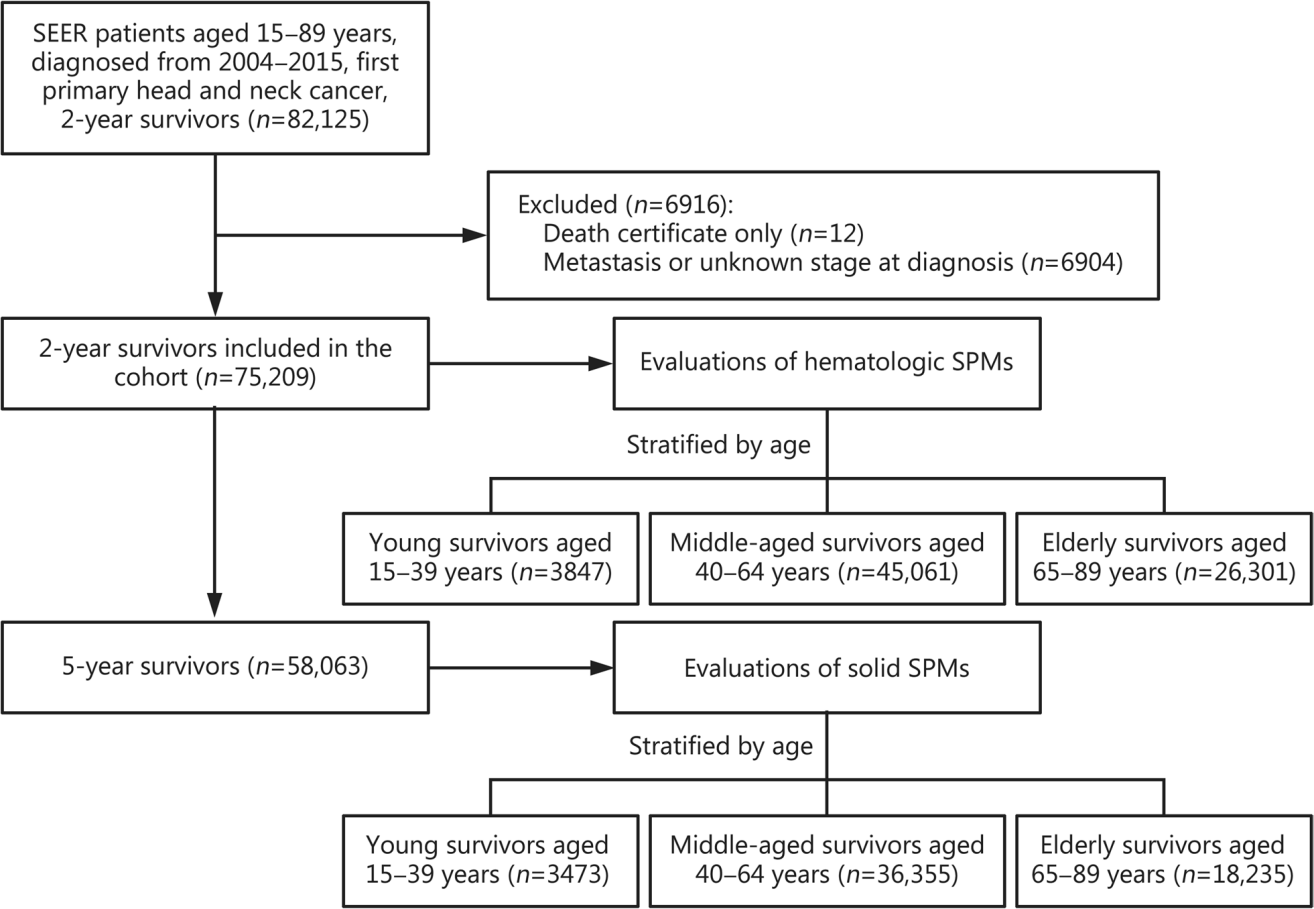
Cohort selection and analysis flowchart for SEER patients aged 15−89 years with FPHNC (2004−2015). SEER Surveillance, Epidemiology, and End Results, SPMs second primary malignancies, FPHNC first primary head and neck cancer

The observed and expected numbers of SPMs were derived using SEER data. Expected counts were calculated by applying age-, sex-, calendar year-, and race-stratified incidence rates from the reference population [24]. All data were expressed with mean ± standard deviation (SD) for normally distributed continuous variables or median (interquartile range, IQR) for non-normally distributed variables, with normality assessed using the Kolmogorov–Smirnov test. Categorical variables are expressed as *n* (%). Only cases with complete data on age (15−89 years) and primary cancer diagnosis (FPHNC) were included. Cases with missing key variables (e.g., metastatic stage) were excluded (Fig. 1). Missing data for other variables (e.g., race, FPHNC stage) were not imputed, as these variables were not included as covariates in the model. No missing data were present in the final analytical dataset.

#### Primary analysis

Primary analysis focused on the association between radiotherapy and SPMs, incorporating cumulative incidence, internal comparison (within the cohort), and external comparison (against the general population). The primary stratification variables for all these three analyses included age groups (15–39, 40–64, and 65–89 years), which were selected based on their alignment with global cancer burden trends for young adults [3] and elderly populations [25], as well as sex (males vs. females). The variable “age (as a continuous variable)” was excluded from the covariates in stratified models to avoid collinearity issues. The variable “sex” was excluded from the covariates in stratified models to avoid redundancy and ensure independent evaluation of their effects. Descriptive analyses with 95% CIs were used to compare groups.

*Cumulative incidence* Fine-Gray competing risk models were used to estimate the cumulative incidence of solid and hematologic SPMs following FPHNC diagnosis, with radiotherapy exposure (whether or not the patient received radiotherapy) serving as the primary grouping variable. These models accounted for two competing risks: 1) death before SPM occurrence, and 2) development of a different SPM type (i.e., solid SPMs were treated as competing events for hematologic SPMs, and hematologic SPMs for solid SPMs in separate analyses). In the SEER database, chemotherapy is categorized as “yes” or “none/unknown”, with the latter indicating no evidence of chemotherapy in medical records. Cancer-directed surgery is categorized as “performed” or “none/unknown”, with the latter indicating no evidence of surgery in medical records. Multivariable Fine-Gray models were adjusted for the following covariates: sex (male vs. female), age (as a continuous variable) at diagnosis of FPHNC, FPHNC site (Additional file 1: Table S3), FPHNC histology (Additional file 1: Tables S4 and S5), cancer-directed surgery (performed vs. none/unknown), and chemotherapy (yes vs. none/unknown). Collinearity diagnostics indicated no concerning multicollinearity [all variance inflation factors (VIFs) < 2], supporting the independence of covariates in the model (Additional file 1: Table S6). Subdistribution hazard ratios (SHRs) were subsequently calculated to evaluate these associations.

*Internal comparison* Internal comparison refers to the primary exposure-outcome analysis within the study cohort, where incidence rate ratios (IRRs) for SPMs were compared between radiotherapy-exposed and unexposed groups. This analysis was conducted using multivariable modified Poisson regression [26] adjusted for sex (male vs. female), age at FPHNC diagnosis, chemotherapy status (yes vs. none/unknown), and latency period (time between FPHNC and SPM diagnoses). All models accounted for potential confounding through covariate adjustment, with collinearity diagnostics confirming variable independence (all VIFs < 1.5) (Additional file 1: Table S7). Model adequacy was verified using Wald tests for parameters and Pearson *χ*^2^ tests for goodness-of-fit.

*External comparison* In this study, external comparison refers to the evaluation of cancer risk in radiotherapy-exposed and unexposed patients relative to the general population, quantified by standardized incidence ratios (SIRs). The SIRs were computed as the ratio of observed cancers in the cohort to expected cases derived from sex-, age-, and calendar year-matched general population incidence rates. To address potential confounding by established risk factors, stratified analyses compared SIRs for: 1) smoking-associated malignancies vs. non-associated malignancies; 2) alcohol-associated malignancies vs. non-associated malignancies; 3) Epstein-Barr virus (EBV)-related cancers vs. EBV-unrelated cancers; and 4) human papillomavirus (HPV)-related cancers vs. HPV-unrelated cancers. This approach contextualizes the cohort’s excess risk while accounting for population-level differences in exposure prevalence.

#### Secondary analyses

For secondary analyses, we compared the cumulative incidence of SPMs between two treatment groups: patients receiving combined radiotherapy and chemotherapy vs. patients receiving radiotherapy alone. This comparison was performed using multivariable Fine-Gray competing risk models, which were adjusted for the following covariates: sex (male vs. female), age (as a continuous variable) at diagnosis of FPHNC, FPHNC site (Additional file 1: Table S3), FPHNC histology (Additional file 1: Tables S4 and S5), and cancer-directed surgery (performed vs. none/unknown).

#### Software and significance level

All analyses were performed using SEER*Stat 8.4.4 [Multiple Primary-Standardized Incidence Ratios (MP-SIR) session], SPSS 26.0, and R 4.4.0, with a significance threshold defined as* P* < 0.05. Figures were prepared using Visio Professional Preview 2024 and Adobe Photoshop 23.0.0.

## Results

The cohort selection process and analysis are summarized in Fig. 1. There were 82,125 2-year FPHNC survivors aged 15−89, diagnosed between 2004 and 2015. After excluding those with death certificates only or with metastasis or unknown stage at diagnosis, 75,209 2-year FPHNC survivors were included in the evaluation of hematologic SPMs. Among these patients, 58,063 survived for 5 years. These 5-year FPHNC survivors were used for the evaluation of solid SPMs.

### Solid SPMs

#### Cumulative incidence

**Fig. 2:**
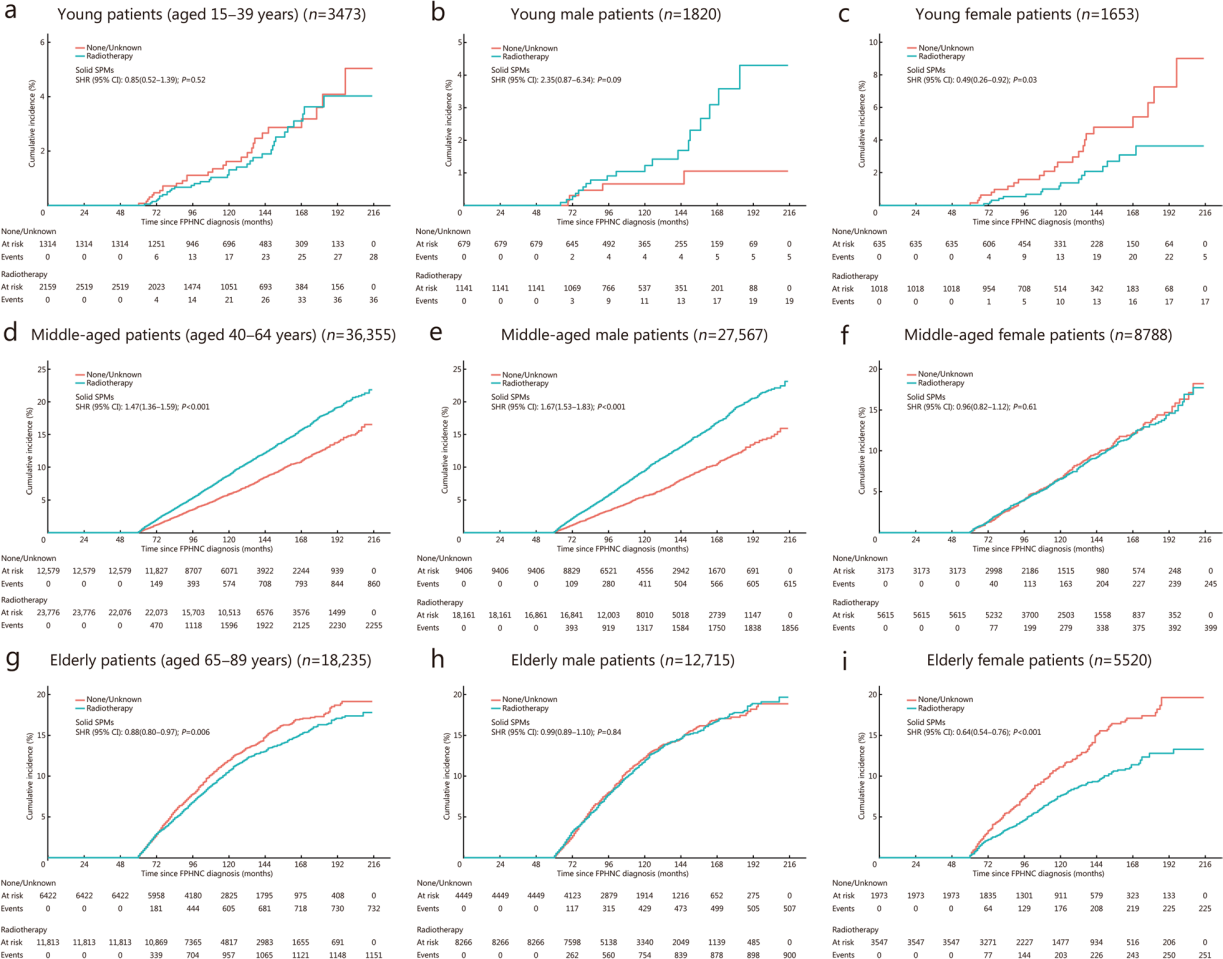
Age- and sex-stratified association between radiotherapy and cumulative incidence of solid SPMs. **a** No significant association between radiotherapy and the cumulative incidence of solid SPMs among young 5-year survivors of FPHNC. **b** No significant association between radiotherapy and the cumulative incidence of solid SPMs among young male patients. **c** Radiotherapy was associated with a lower risk of developing solid SPMs (SHR = 0.49, 95% CI 0.26−0.92; *P* = 0.030) among young female patients. **d** Among middle-aged patients, radiotherapy was associated with a higher risk of developing solid SPMs (SHR = 1.47, 95% CI 1.36−1.59;* P* < 0.001). **e** Among middle-aged male patients, radiotherapy was associated with a higher risk of developing solid SPMs (SHR = 1.67, 95% CI 1.53−1.83;* P* < 0.001). **f** No significant association between radiotherapy and the cumulative incidence of solid SPMs among middle-aged female patients. **g** Radiotherapy was associated with a lower risk of developing solid SPMs (SHR = 0.88, 95% CI 0.80−0.97;* P* = 0.006) among elderly patients. **h** No significant association between radiotherapy and the cumulative incidence of solid SPMs among elderly male patients. **i** Radiotherapy was associated with a lower risk of developing solid SPMs (SHR = 0.64, 95% CI 0.54−0.76;* P* < 0.001) among elderly female patients. Multivariable Fine-Gray models adjusted for sex, FPHNC site, FPHNC histology, cancer-directed surgery, and chemotherapy (**a, d, g**), and adjusted for FPHNC site, FPHNC histology, cancer-directed surgery, and chemotherapy (sex excluded due to stratification) (**b, c, e, f, h, i**). CI confidence interval, FPHNC first primary head and neck cancer, SHR subdistribution hazard ratio, SPMs second primary malignancies

Among the 58,063 5-year survivors, 37,748 (65.0%) received radiotherapy, and 5062 solid SPMs (excluding HNC) were observed during the follow-up period (Additional file 1: Table S8). Multivariable Fine-Gray’s competing risk analysis indicated a higher risk of developing solid SPMs in patients who received radiotherapy (SHR = 1.20, 95% CI 1.13−1.27;* P* < 0.001; Additional file 1: Fig. S1a). No significant association was observed for young patients (aged 15–39 years) (Fig. 2a). A significantly higher risk of developing solid SPMs was found among middle-aged patients (aged 40–64 years) who received radiotherapy (SHR = 1.47, 95% CI 1.36−1.59;* P* < 0.001; Fig. 2d), while a lower risk was identified among elderly patients (aged 65–89 years) (SHR = 0.88, 95% CI 0.80−0.97;* P* = 0.006; Fig. 2g). These associations varied by sex. When stratified by sex, radiotherapy was associated with a lower risk of developing solid SPMs (SHR = 0.49, 95% CI 0.26−0.92; *P* = 0.03) among young female patients (Fig. 2c). In contrast, no significant association between radiotherapy and the cumulative incidence of solid SPMs was identified among young male patients (Fig. 2b). Radiotherapy was associated with a higher risk of developing solid SPMs (SHR = 1.67, 95% CI 1.53−1.83;* P* < 0.001) among middle-aged male patients (Fig. 2e), while no significant association was found between radiotherapy and the cumulative incidence of solid SPMs among middle-aged female patients (Fig. 2f). Radiotherapy was associated with a lower risk of developing solid SPMs (SHR = 0.64, 95% CI 0.54−0.76;* P* < 0.001) among elderly female patients (Fig. 2i), while no significant association was identified between radiotherapy and the cumulative incidence of solid SPMs among elderly male patients (Fig. 2h).

Among the 5-year survivors who received radiotherapy, 15,217 (40.3%, the analysis set was the 37,748 patients who received radiotherapy) also received chemotherapy. Patients treated with both radiotherapy and chemotherapy had a significantly higher risk of developing solid SPMs compared to those who received radiotherapy alone, as determined by multivariable Fine-Gray’s competing risk models (SHR = 1.75, 95% CI 1.61−1.91; *P* < 0.001; Additional file 1: Fig. S2a). This trend was consistent across different age groups (Additional file 1: Fig. S2b-d).

#### Internal comparison

**Fig. 3:**
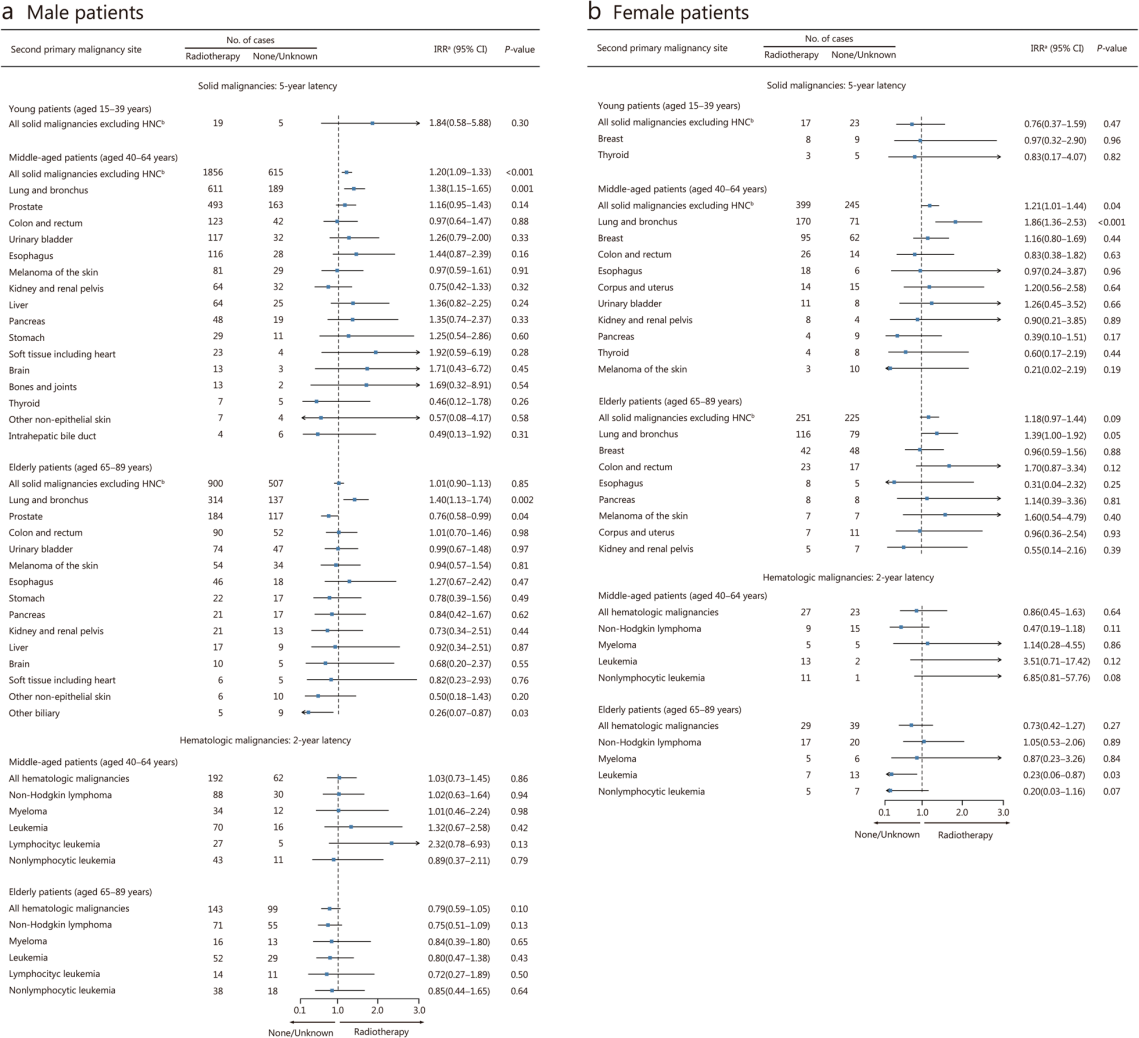
Age- and sex-stratified IRRs and 95% CIs for SPMs associated with radiotherapy in FPHNC. IRRs and 95% CIs for SPMs associated with radiotherapy among male (**a**) and female (**b**) patients. ^a^Modified Poisson regression models, adjusted for sex, latency (time between FPHNC and SPM diagnoses), and chemotherapy. ^b^The category of all solid malignancies includes the ones listed in this figure as well as those malignancy sites with < 10 events. CI confidence interval, HNC head and neck cancer, IRR incidence rate ratio, FPHNC first primary head and neck cancer, SPMs second primary malignancies

Among the 5-year survivors, radiotherapy was associated with an increased risk of solid SPMs (excluding HNC) (IRR = 1.16, 95% CI 1.08−1.24;* P* < 0.001; Additional file 1: Fig. S3). Among the solid SPMs, radiotherapy was associated with a higher risk of SPMs in the lung/bronchus (IRR = 1.49, 95% CI 1.33−1.68;* P* < 0.001; Additional file 1: Fig. S3). For young patients, SPM sites with more than 10 events were limited to breast and thyroid (Additional file 1: Fig. S4). Radiotherapy was associated with an increased risk of solid SPMs in middle-aged patients (IRR = 1.21, 95% CI 1.11−1.32; *P* < 0.001; Additional file 1: Fig. S4). Radiotherapy was associated with an elevated risk of SPMs in the lung/bronchus in both middle-aged patients (IRR = 1.51, 95% CI 1.28−1.76;* P* < 0.001) and elderly patients (IRR = 1.42, 95% CI, 1.19−1.70;* P* < 0.001; Additional file 1: Fig. S4). When the analysis was further stratified by sex, radiotherapy was associated with an elevated risk of SPMs in the lung/bronchus in both male and female patients (Fig. 3).

#### External comparison

For patients who received radiotherapy, the highest SIR (SIR = 27.50, 95% CI 12.57−52.20) was observed in second primary tracheal malignancies, followed by bones/joints, esophagus, lung/bronchus, soft tissue including the heart, liver urinary bladder, and colon/rectum (Additional file 1: Table S9). In all three age groups, radiotherapy was significantly associated with increased risks of solid SPMs (Additional file 1: Tables S10-S12). When the analysis was further stratified by sex, radiotherapy was associated with a higher risk of developing solid SPMs among young male patients, but not among young female patients (Additional file 1: Table S13). This result was consistent with that of Fine-Gray’s competing risk analysis. Among middle-aged patients who received radiotherapy, the highest SIR (SIR = 7.72, 95% CI 4.32−12.73) was observed in SPMs in the bones/joints, followed by esophagus, lung/bronchus, vulva, soft tissue including the heart, urinary bladder, and liver (Additional file 1: Table S11). For elderly patients, the highest SIR (SIR = 3.87, 95% CI 2.91−5.05) was observed for second primary esophageal malignancies, followed by lung/bronchus and colon/rectum (Additional file 1: Table S12).

### Hematologic SPMs

#### Cumulative incidence

**Fig. 4:**
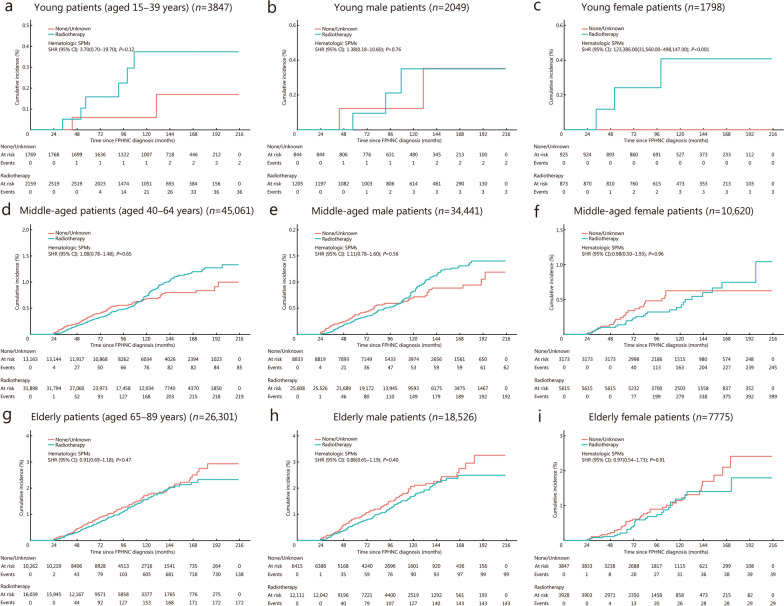
Age- and sex-stratified association between radiotherapy and cumulative incidence of hematologic SPMs. **a** No significant association between radiotherapy and the cumulative incidence of hematologic SPMs among young 2-year survivors of FPHNC. **b** No significant association between radiotherapy and the cumulative incidence of hematologic SPMs among young male patients. **c** Radiotherapy was associated with a higher risk of developing hematologic SPMs (SHR = 123,386.00, 95% CI 31,560.00−498,147.00;* P* < 0.001) among young female patients. **d** No significant association between radiotherapy and the cumulative incidence of hematologic SPMs among middle-aged patients. **e** No significant association between radiotherapy and the cumulative incidence of hematologic SPMs among middle-aged male patients. **f** No significant association between radiotherapy and the cumulative incidence of hematologic SPMs among middle-aged female patients. **g** No significant association between radiotherapy and the cumulative incidence of hematologic SPMs among elderly patients. **h** No significant association between radiotherapy and the cumulative incidence of hematologic SPMs among elderly male patients. **i** No significant association between radiotherapy and the cumulative incidence of hematologic SPMs among elderly female patients. Multivariable Fine-Gray models adjusted for sex, FPHNC site, FPHNC histology, cancer-directed surgery, and chemotherapy (**a, d, g**), and adjusted for FPHNC site, FPHNC histology, cancer-directed surgery, and chemotherapy (sex excluded due to stratification) (**b, c, e, f, h, i**). CI confidence interval, FPHNC first primary head and neck cancer, SHR subdistribution hazard ratio, SPMs second primary malignancies

Among the 75,209 2-year survivors, 50,015 (66.5%) underwent radiotherapy, and 622 (0.8%) were diagnosed with hematologic SPMs during follow-up (Additional file 1: Table S14). Multivariable Fine-Gray’s competing risk analysis revealed no significant association between radiotherapy and the cumulative incidence of hematologic SPMs in all three age groups (Fig. 4; Additional file 1: Fig. S1b).

Among the 2-year survivors who received radiotherapy, 28,967 (57.9%, the analysis set was the 50,015 patients who underwent radiotherapy) also underwent chemotherapy. No significant difference in the risk of developing hematologic SPMs was observed between patients who underwent radiotherapy alone and those treated with a combination of radiotherapy and chemotherapy, according to multivariable Fine-Gray’s competing risk analysis (Additional file 1: Fig. S5).

#### Internal comparison

Among the 75,209 patients in the cohort, modified Poisson regression analysis identified no significant overall decrease in the risk of hematologic SPMs associated with radiotherapy (Additional file 1: Fig. S3). However, for elderly patients, radiotherapy was associated with a decreased risk for hematologic SPMs (IRR = 0.77, 95% CI 0.60−0.99;* P* = 0.045; Additional file 1: Fig. S4).

#### External comparison

After radiotherapy, the incidence of second primary non-lymphocytic leukemia was higher than expected (SIR = 1.41, 95% CI 1.14−1.72), whereas the incidence among patients without radiotherapy was lower than expected (Additional file 1: Table S15). The incidence of second primary non-Hodgkin lymphoma was notably higher than expected in young patients who received radiotherapy (SIR = 4.01, 95% CI 1.47−8.74; Additional file 1: Table S16). For middle-aged patients, the risk of second primary non-lymphocytic leukemia was significantly elevated compared to the general population (SIR = 1.63, 95% CI 1.23−2.13; Additional file 1: Table S16). For elderly patients, radiotherapy was significantly associated with reduced risks of second primary myeloma (SIR = 0.57, 95% CI 0.35−0.87) and second primary lymphocytic leukemia (SIR = 0.49, 95% CI 0.28−0.79; Additional file 1: Table S16).

## Discussion

This SEER-based study investigated the association between radiotherapy and the development of SPMs in HNC patients. An age-dependent association was revealed in the findings. Specifically, increased risks of solid SPMs were observed for middle-aged patients, while decreased risks for hematologic SPMs were identified in elderly patients. These observations highlight the importance of considering age as a factor in treatment decisions and long-term surveillance for HNC patients.

Evaluation of SIR provides valuable preliminary data at the population level to support research on the risk of SPMs relative to the general US population [24, 27]. Age-stratified SIRs of SPMs were identified in the present study. The radiotherapy-associated risk of second primary non-Hodgkin lymphoma was elevated in young patients. Among middle-aged patients treated with radiotherapy, higher risk ratios for SPMs were observed in the bones/joints, esophagus, lung/bronchus, vulva, soft tissue including the heart, urinary bladder, liver, and non-lymphocytic leukemia. For elderly patients receiving radiotherapy, higher risks for SPMs were identified in the esophagus, lung/bronchus, and colon/rectum. The occurrence of SPMs outside the primary field of irradiation may be explained by mechanisms such as the bystander effect, which affects non-irradiated neighboring cells, as well as the abscopal effect, which affects more distant cells [13, 28, 29]. The bystander effect involves the transmission of signals from irradiated cells to nearby non-irradiated cells that result in DNA damage and other cellular changes. Conversely, the abscopal effect is a phenomenon where immune-mediated responses triggered by localized radiotherapy result in anti-tumor effects at distant, non-irradiated sites [28, 29]. Although the abscopal effect is traditionally viewed as an anti-tumor mechanism, it may also provide insights into the development of distant SPMs following local radiotherapy for HNCs. By inducing systemic immune modulation, genomic instability, and chronic inflammation, radiotherapy may create a microenvironment that promotes carcinogenesis at distant sites [30–32]. Immunotherapy such as immune checkpoint inhibitors has the potential to enhance the anti-tumor effects of radiotherapy through mechanisms such as the abscopal effect [33]. While immunotherapy may reduce the risk of SPMs by enhancing immune surveillance, it may also contribute to SPM development through chronic inflammation or immune dysregulation [30–32]. The long-term impact of combining radiotherapy and immunotherapy on SPM risk in HNC patients remains uncertain. This uncertainty highlights the need for further research.

The methodology used in this study follows the guidelines described in the SEER monograph, which emphasizes the distinction between observed and expected rates of SPMs [24]. By comparing SIRs for patients with or without radiotherapy, the analysis sought to identify any excess risk attributable to treatment. However, it is acknowledged that these analyses cannot definitively establish causality on an individual level.

Patients with HNC exhibit unique characteristics compared to the general population. These characteristics include differences in lifestyle, smoking habits, alcohol use, HPV infections, EBV infections, and genetic predispositions, that may confound the results of external comparisons [1, 2, 34, 35]. Confounding by smoking may have biased the SIRs for smoking-associated cancers (e.g., lung/bronchus, colon/rectum, and urinary bladder) in a negative direction [36, 37]. Likewise, confounding by alcohol may have biased the SIRs for alcohol-associated cancers (e.g., liver and esophagus) in the negative direction [37, 38]. Confounding by HPV infections may have biased the SIRs for HPV-associated cancers (e.g., anal cancer) in the negative direction [39, 40]. Confounding by EBV infections may have biased the SIRs for EBV-associated cancers (e.g., gastric cancer) in the negative direction [41, 42].

The internal comparison conducted in this study represents a significant improvement over prior studies [43–46]. This approach minimized confounding from smoking and alcohol consumption, as these factors did not influence the decision to administer radiotherapy in HNC patients [16]. Through these internal comparisons, an increased risk of solid SPMs was observed in middle-aged patients. Comparing the present findings with those from other cancer studies is challenging due to variations in cohort inclusion criteria, and the wide range of clinical and sociodemographic factors that influence outcomes [47]. Nonetheless, the findings of the present study are consistent with those reported by Hashibe et al. [13], who identified an increased risk of solid SPMs associated with radiotherapy in HNC patients.

According to our study, males were more likely to develop solid SPMs compared to females, a finding consistent with previous studies [13, 48]. The increased sensitivity of males to radiation-induced damage likely results from a combination of weaker DNA repair mechanisms, hormonal differences, and immune system variations [49, 50]. Further research is needed to fully understand these mechanisms and develop targeted strategies to mitigate SPM risk in male patients undergoing radiotherapy.

Apart from radiotherapy, chemotherapy is frequently used in the management of HNC. Chemotherapy has also been identified as a significant risk factor for SPMs, particularly for hematologic SPMs [51, 52]. Therefore, all multivariable models in this study were adjusted for chemotherapy to ensure more robust results. Among elderly patients, radiotherapy was associated with a decreased risk of hematologic SPMs in the internal comparison, contrary to earlier findings from SEER-based studies [11, 13]. Previous SEER-based studies did not account for the potential effect of chemotherapy, as data were not included in the earlier publicly accessible SEER database [13, 16]. This could have accounted for the difference between their findings and the results of the present study. Our results have also indicated that patients who received a combination of radiotherapy and chemotherapy had a higher risk of developing solid SPMs compared to those treated with radiotherapy alone, especially in young patients. In clinical practice, the concurrent use of radiotherapy and chemotherapy for the treatment of HNC should be approached with caution, particularly in young patients.

Smoking-associated HNCs, typically diagnosed in elderly patients, are slowly declining globally. This is due, in part, to reduced tobacco use [34]. In contrast, cases of HPV-associated oropharyngeal cancer are on the rise, especially among younger adults in Northern Europe and North America. These trends reflect the long latency period of HPV-induced carcinogenesis, which often occurs 10 to 30 years after initial exposure [34, 39, 40]. The observed differences in solid SPM risk among younger, middle-aged, and elderly patients following radiotherapy likely stem from a complex interplay of factors. Young patients may not have lived long enough to accumulate significant environmental risk factors or for SPMs to manifest, and their cancer risk may be driven more by genetic predisposition [53, 54]. In contrast, elderly patients may develop HNC due to age-related biological degradation but have a lower post-radiotherapy SPM risk due to competing risks, shorter lifespans, and potentially less aggressive treatment [55–57]. Middle-aged patients appear to have the highest SPM risk due to a combination of lifestyle-related exposures, a sufficient lifespan for SPM development, and cumulative cellular damage [37, 58, 59]. Further research is needed to disentangle these contributing factors and clarify the role of radiotherapy in SPM development among different age groups. Understanding these dynamics can help tailor cancer treatment and surveillance strategies to minimize SPM risk and optimize patient treatment outcomes.

This study reveals age-stratified associations between radiotherapy and SPMs, underscoring the need for more age-specific treatment protocols in HNC management. Implementing such protocols enables a more tailored approach that balances the benefits of radiotherapy with the potential risks of developing SPMs. From a patient care perspective, these findings highlight the importance of individualized treatment planning, informed consent, and long-term surveillance [60]. Discussions with patients should include the potential risks and benefits of radiotherapy, ensuring they are well-informed before making treatment decisions.

The present study has significant strengths. First, it utilizes a large-scale, population-based database that provides a robust reflection of real-world associations in a community setting. The extended follow-up period enables reliable estimates of the long-term outcomes associated with radiotherapy. A major aspect of the methodology was the focused comparison within the cohort, with separate evaluations conducted for young, middle-aged, and elderly patients. This approach revealed the varied relationships between radiotherapy and the risks of developing SPMs among different age groups.

The present study also has several limitations. Although the SEER database is comprehensive, it lacks specific details on radiotherapy fields, doses, and techniques, as well as information on treatment administered during relapse. This limitation may result in underestimating the actual use of radiotherapy, cancer-directed surgery, and chemotherapy [24]. Contemporary radiotherapy techniques such as intensity-modulated radiotherapy (IMRT), volumetric modulated arc therapy (VMAT), and cone-beam computed tomography (CBCT)-guided radiotherapy have revolutionized cancer treatment by improving precision and reducing toxicity. However, their impact on SPM risk remains incompletely understood. Indeed, IMRT and VMAT enable highly conformal dose delivery, sparing surrounding normal tissues and reducing the volume of irradiated healthy tissue. This precision theoretically lowers the risk of SPMs by minimizing radiation exposure to non-target tissues. However, these techniques often expose a larger volume of non-target tissues to low-dose radiation, compared to older techniques such as three-dimensional conformal radiotherapy. This “low-dose bath” effect may increase the risk of SPMs in distant tissues, as even low doses of radiation can induce DNA damage and carcinogenesis [61, 62]. Modern techniques may also generate more scattered radiation to distant sites, further contributing to SPMs outside the treatment field [61]. By improving precision, CBCT-guided radiotherapy may lower the risk of acute and late toxicities [62]. However, the additional imaging dose from CBCT, albeit small, may cumulatively contribute to SPM risk over time [63]. Radiotherapy doses as high as 66−74 Gy are often used in high-risk HNCs such as advanced-stage tumors, positive margins, or extracapsular extension [1]. These higher doses may increase the risk of SPMs due to greater radiation exposure and DNA damage in normal tissues [8]. In contrast, intermediate-risk HNCs are usually treated with lower doses (50−60 Gy) [1]. While this reduces the risk of acute toxicity, the long-term risk of SPMs remains a concern, particularly in young and middle-aged patients with longer life expectancy. The association in young patients should be interpreted with caution due to the wide CI and small sample size, as seen in the data presented in Additional file 1: Fig. S2b. Long-term studies are needed to evaluate the impact of these contemporary radiotherapy techniques on SPM risk, particularly in younger HNC patients. Future research should focus on establishing dose–response relationships for SPMs, taking into account radiotherapy dose, fractionation, and treatment volumes.

Furthermore, the SEER database may not fully represent all demographic groups, particularly those with different levels of healthcare access [64]. Consequently, these findings should be interpreted with caution when applying them to broader populations. Future studies with more detailed and comprehensive data collection are necessary to validate and expand upon these results.

## Conclusions

This large population-based study demonstrated that radiotherapy was associated with increased risks of SPMs in survivors of non-metastatic primary HNC. Furthermore, an age-stratified association between radiotherapy and SPMs was identified. Middle-aged patients (aged 40–64 years) may have an elevated risk of developing SPMs as a result of radiotherapy. Therefore, the use of radiotherapy in this age group should be approached with caution. This study highlights the importance of age-tailored treatment decisions and long-term surveillance to reduce SPM risks in vulnerable age groups among HNC survivors.

## Supplementary Information

**Additional file 1. Table S1** Site recode ICD-O-3 2023 revision. **Table S2** Site recode ICD-O-3/WHO 2008 definition (for SIRs). **Table S3** Site of FPHNC among 2- and 5-year survivors [*n* (%)]. **Table S4** Histology recodes of FPHNC among different age groups of 5-year survivors based on histologic type ICD-O-3 [*n* (%)]. **Table S5** Histology recodes of FPHNC among different age groups of 2-year survivors based on Histologic Type ICD-O-3 [*n* (%)]. **Table S6** Collinearity diagnostics for multivariable Fine-Gray competing risk models. **Table S7** Collinearity diagnostics for multivariable modified Poisson regression models. **Table S8** Baseline characteristics of the 5-year survivors of FPHNC. **Table S9** SIRs for solid SPMs: external comparison with the general population by radiotherapy exposure, and IRRs: internal comparison within the cohort by radiotherapy exposure, in 5-year survivors of FPHNC. **Table S10** SIRs for solid SPMs, in young 5-year survivors of FPHNC: external comparison with the general population by radiotherapy exposure. **Table S11** SIRs for solid SPMs, in middle-aged 5-year survivors of FPHNC: external comparison with the general population by radiotherapy exposure.** Table S12** SIRs for solid SPMs, in elderly 5-year survivors of FPHNC: external comparison with the general population by radiotherapy exposure. **Table S13** Age- and sex-stratified SIRs for SPMs, in survivors of FPHNC: external comparison with the general population by radiotherapy exposure. **Table S14** Baseline characteristics of the 2-year survivors of FPHNC. **Table S15** SIRs for hematologic SPMs: external comparison with the general population by radiotherapy exposure, and IRRs: internal comparison within the cohort by radiotherapy exposure in 2-year survivors of FPHNC. **Table S16** Age-stratified SIRs for hematologic SPMs, in 2-year survivors of FPHNC: external comparison with the general population by radiotherapy exposure. **Fig. S1** Associations between radiotherapy and cumulative incidence of SPMs. **Fig. S2** Associations between chemotherapy and cumulative incidence of solid SPMs among 5-year survivors treated with radiotherapy. **Fig. S3** IRRs and 95% CIs for solid and hematologic SPMs associated with radiotherapy in FPHNC. **Fig. S4** Age-stratified IRRs and 95% CIs for SPMs associated with radiotherapy in FPHNC. **Fig. S5** Associations between chemotherapy and cumulative incidence of hematologic SPMs among 2-year survivors treated with radiotherapy.

## Data Availability

The datasets used and analyzed during the current study are publicly available from the SEER (https://seer.cancer.gov) based on the SEER official guidelines.

## Abbreviations

AJCC: The American Joint Committee on Cancer
CBCT: Cone-beam computed tomography
CI: Confidence interval
EBV: Epstein-Barr virus
FPHNC: First primary head and neck cancer
HNC: Head and neck cancer
HPV: Human papillomavirus
ICD-O: International Classification of Diseases for Oncology
IMRT: Intensity-modulated radiotherapy
IQR: Interquartile range
IRR: Incidence rate ratio
LA: Los Angeles
MP-SIR: Multiple Primary-Standardized Incidence Ratios
MSA: Metropolitan Statistical Area
NCCN: National Comprehensive Cancer Network
SD: Standard deviation
SEER: Surveillance, Epidemiology, and End Results
SF: San Francisco
SHR: Subdistribution hazard ratio
SIR: Standardized incidence ratio
SJM: San Jose-Monterey
SPM: Second primary malignancy
STROBE: Strengthening the Reporting of Observational Studies in Epidemiology
TNM: Tumor, Node, and Metastasis
VIFs: Variance inflation factors
VMAT: Volumetric modulated arc therapy
