# Supplementary material for: Age-stratified associations between radiotherapy and SPMs for FPHNC: a population-based cohort study
Source: Mil Med Res. 2025 May 21;12:22. doi: 10.1186/s40779-025-00612-4 (PMC12093708; doi:10.1186/s40779-025-00612-4)
Supplement: Supplementary file 1 — Additional file 1. Table S1 Site recode ICD-O-3 2023 revision. Table S2 Site recode ICD-O-3/WHO 2008 definition (for SIRs). Table S3 Site of FPHNC among 2- and 5-year survivors [n (%)]. Table S4 Histology recodes of FPHNC among different age groups of 5-year survivors based on histologic type ICD-O-3 [n (%)]. Table S5 Histology recodes of FPHNC among different age groups of 2-year survivors based on Histologic Type ICD-O-3 [n (%)]. Table S6 Collinearity diagnostics for multivariable Fine-Gray competing risk models. Table S7 Collinearity diagnostics for multivariable modified Poisson regression models. Table S8 Baseline characteristics of the 5-year survivors of FPHNC. Table S9 SIRs for solid SPMs: external comparison with the general population by radiotherapy exposure, and IRRs: internal comparison within the cohort by radiotherapy exposure, in 5-year survivors of FPHNC. Table S10 SIRs for solid SPMs, in young 5-year survivors of FPHNC: external comparison with the general population by radiotherapy exposure. Table S11 SIRs for solid SPMs, in middle-aged 5-year survivors of FPHNC: external comparison with the general population by radiotherapy exposure. Table S12 SIRs for solid SPMs, in elderly 5-year survivors of FPHNC: external comparison with the general population by radiotherapy exposure. Table S13 Age- and sex-stratified SIRs for SPMs, in survivors of FPHNC: external comparison with the general population by radiotherapy exposure. Table S14 Baseline characteristics of the 2-year survivors of FPHNC. Table S15 SIRs for hematologic SPMs: external comparison with the general population by radiotherapy exposure, and IRRs: internal comparison within the cohort by radiotherapy exposure in 2-year survivors of FPHNC. Table S16 Age-stratified SIRs for hematologic SPMs, in 2-year survivors of FPHNC: external comparison with the general population by radiotherapy exposure. Fig. S1 Associations between radiotherapy and cumulative incidence of SPMs. Fig. S2 Associations be [file 40779_2025_612_MOESM1_ESM.pdf]

Table S1 Site recode ICD-O-3 2023 revision

Head and neck	ICD-O-3 site	ICD-O-3 histology (type)	ICD-O-3 behavior	Recode value
Lip	C000 – C006, C008 – C009	Excluding 9050 – 9055, 9140, 9590 – 9993	2 – 3	001
Tongue anterior	C020 – C023, C028 – C029			002
Gum	C030 – C031, C039, C062			003
Floor of mouth	C040 – C041, C048 – C049			004
Palate excluding soft and uvula	C050			005
Buccal mucosa	C060, C061			006
Mouth other	C058 – C059, C068 – C069			007
Major salivary glands	C079, C080 – C081, C088 – C089			008
Nasopharynx	C110 – C113, C118 – C119			009
Oropharynx	C019, C024, C051 – C052, C090 – C091, C098 – C099, C100, C102 – C104, C108 – C109			010
Hypopharynx	C129, C130 – C132, C138 – C139			011
Pharynx and oral cavity other	C140, C142, C148			012
Nasal cavity and paranasal sinuses	C300, C310, C311			013
Sinus other	C301, C312 – C313, C318 – C319			014
Larynx	C101, C320 – C323, C328 – C329			015

ICD-O-3 International Classification of Diseases for Oncology-3

Table S2 Site recode ICD-O-3/WHO 2008 definition (for SIRs)

Group	ICD-O-3 site	ICD-O-3 histology (type)	Recode value
Digestive system			
Esophagus	C150 – C159	Excluding 9050 – 9055, 9140, 9590 – 9993	21010
Stomach	C160 – C169		21020
Small intestine	C170 – C179		21030
Colon and rectum			
Colon excluding rectum			
Cecum	C180	Excluding 9050 – 9055, 9140, 9590 – 9993	21041
Appendix	C181		21042
Ascending colon	C182		21043
Hepatic flexure	C183		21044
Transverse colon	C184		21045
Splenic flexure	C185		21046
Descending colon	C186		21047
Sigmoid colon	C187		21048
Largeintestine, NOS	C188 – C189, C260		21049
Rectum and rectosigmoid junction			
Rectosigmoid junction	C199	Excluding 9050 – 9055, 9140, 9590 – 9993	21051
Rectum	C209		21052
Anus, anal canal, and anorectum	C210 – C212, C218		21060

Group	ICD-O-3 site	ICD-O-3 histology (type)	Recode value
Liver and intrahepatic bile duct			
Liver	C220	Excluding 9050 – 9055, 9140, 9590 – 9993	21071
Intrahepatic bile duct	C221		21072
Gallbladder	C239		21080
Other biliary	C240 – C249		21090
Pancreas	C250 – C259		21100
Retroperitoneum	C480		21110
Peritoneum, omentum, and mesentery	C481 – C482		21120
Other digestive organs	C268 – C269, C488		21130
Respiratory system			
Lung and bronchus	C340 – C349	Excluding 9050 – 9055, 9140, 9590 – 9993	22030
Pleura	C384		22050
Trachea, mediastinum, and other respiratory Organs	C339, C381 – C383, C388, C390, C398, C399		22060
**Bones and joints**	C400 – C419	Excluding 9050 – 9055, 9140, 9590 – 9993	23000
**Soft tissue including heart**	C380, C470 – C479, C490 – C499	Excluding 9050 – 9055, 9140, 9590 – 9993	24000
Skin excluding basal and squamous			
Melanoma of the skin	C440 – C449	8720 – 8790	25010
Other non-epithelial skin	C440 – C449	Excluding 8000 – 8005, 8010 – 8046, 8050 – 8084, 8090 – 8110, 8720 – 8790, 9050 – 9055, 9140, 9590 – 9993	25020
**Breast**	C500 – C509	Excluding 9050 – 9055, 9140, 9590 – 9993	26000

Group	ICD-O-3 site	ICD-O-3 histology (type)	Recode value
Female genital system			
Cervix uteri	C530 – C539	Excluding 9050 – 9055, 9140, 9590 – 9993	27010
Corpus and uterus, NOS			
Corpus uteri	C540 – C549	Excluding 9050 – 9055, 9140, 9590 – 9993	27020
Uterus, NOS	C559		27030
Ovary	C569		27040
Vagina	C529		27050
Vulva	C510 – C519		27060
Other female genital organs	C570 – C579, C589		27070
Male genital system			
Prostate	C619	Excluding 9050 – 9055, 9140, 9590 – 9993	28010
Testis	C620 – C629		28020
Penis	C600 – C609		28030
Other male genital organs	C630 – C639		28040
Urinary system			
Urinary bladder	C670 – C679	Excluding 9050 – 9055, 9140, 9590 – 9993	29010
Kidney and renal pelvis	C649, C659		29020
Ureter	C669		29030
Other urinary organs	C680 – C689		29040
Eye and orbit	C690 – C699	Excluding 9050 – 9055, 9140, 9590 – 9993	30000
Brain and other nervous system			

Group	ICD-O-3 site	ICD-O-3 histology (type)	Recode value
Brain	C710 – C719	Excluding 9050 – 9055, 9140, 9530 – 9539, 9590 – 9993	31010
Cranial nerves other nervous system	C710 – C719	9530-9539	31040
	C700 – C709, C720 – C729	Excluding 9050-9055, 9140, 9590-9993	
Endocrine system			
Thyroid	C739	Excluding 9050 – 9055, 9140, 9590 – 9993	32010
Other endocrine including thymus	C379, C740 – C749, C750 – C759		32020
Lymphoma			
Hodgkin lymphoma			
Hodgkin - nodal	C024, C098 – C099, C111, C142, C379, C422, C770 – C779	9650-9667	33011
Hodgkin - extranodal	All other sites		33012
Non-Hodgkin lymphoma			
NHL - nodal	C024, C098, C099, C111, C142, C379, C422, C770 – C779	9590 – 9597, 9670 – 9671, 9673, 9675, 9678 – 9680, 9684, 9687 – 9691, 9695, 9698 – 9702, 9705, 9708 – 9709, 9712, 9714 – 9719, 9724 – 9729, 9735, 9737 – 9738, 9811 – 9818, 9823, 9827, 9837	33041
NHL - extranodal	All sites except C024, C098 – C099, C111, C142, C379, C422, C770 – C779	9590 – 9597, 9670 – 9671, 9673, 9675, 9678 – 9680, 9684, 9687, 9688, 9689 – 9691, 9695, 9698 – 9702, 9705, 9708 – 9709, 9712, 9714 – 9719, 9724 – 9729, 9735, 9737, 9738	33042
	All sites except C024, C098 – C099, C111, C142, C379, C420 – C422, C424, C770 – C779	9811 – 9819, 9823, 9827, 9837	
Myeloma	-	9731 – 9732, 9734	34000
Leukemia			

Group	ICD-O-3 site	ICD-O-3 histology (type)	Recode value
Lymphocytic leukemia			
Acute lymphocytic leukemia	-	9826, 9835 – 9836	35011
	C420, C421, C424	9811 – 9819, 9837	
Chronic lymphocytic leukemia	C420, C421, C424	9823	35012
Other lymphocytic leukemia	-	9820, 9832 – 9834, 9940	35013
Myeloid and monocytic leukemia			
Acute myeloid leukemia	-	9840, 9861, 9865 – 9867, 9869, 9871 – 9874, 9877 – 9879, 9895 – 9897, 9898, 9910 – 9912, 9920	35021
Acute monocytic leukemia	-	9891	35031
Chronic myeloid leukemia	-	9863, 9875 – 9876, 9945 – 9946	35022
Other myeloid/monocytic leukemia	-	9860, 9930	35023
Other leukemia			
Other acute leukemia	-	9801, 9805 – 9809, 9931	35041
Aleukemic, subleukemic, and NOS	-	9733, 9742, 9800, 9831, 9870, 9948, 9963 – 9964	35043
	C420, C421, C424	9827	

ICD-O-3 International Classification of Diseases for Oncology-3, *WHO* World Health Organization, *NOS* not otherwise specified, *SIR* standardized incidence ratio

Table S3 Site of FPHNC among 2- and 5-year survivors [*n* (%)]

**FPHNC site**	**2-year FPHNC survivors (*n* = 75,209)**	**5-year FPHNC survivors (*n* = 58,063)**
Buccal mucosa	1228 (1.63)	932 (1.61)
Floor of mouth	2482 (3.30)	1686 (2.90)
Gum	2323 (3.09)	1660 (2.86)
Hypopharynx	1813 (2.41)	1113 (1.92)
Larynx	17,510 (23.28)	12,734 (21.93)
Lip	4705 (6.26)	3892 (6.70)
Major salivary glands	6281 (8.35)	5086 (8.76)
Mouth other	711 (0.95)	562 (0.97)
Nasal cavity and paranasal sinuses	2228 (2.96)	1668 (2.87)
Nasopharynx	3326 (4.42)	2602 (4.48)
Oropharynx	23,828 (31.68)	19,137 (32.96)
Palate excluding soft and uvula	834 (1.11)	675 (1.16)
Tongue anterior	7940 (10.56)	6316 (10.88)

FPHNC first primary head and neck cancer

Table S4 Histology recodes of FPHNC among different age groups of 5-year survivors based on histologic type ICD-O-3 [*n* (%)]

**Histology recodes**	**All the 5-year survivors (*n* = 58,063)**	**Young patients (aged 15 – 39 years) (*n* = 3473)**	**Middle-aged patients (aged 40 – 64 years) (*n* = 36,355)**	**Elderly patients (aged 65 – 89 years) (*n* = 18,235)**
8000 – 8009: unspecified neoplasms	95 (0.16)	10 (0.29)	53 (0.15)	32 (0.18)
8010 – 8049: epithelial neoplasms	2131 (3.67)	320 (9.21)	1240 (3.41)	571 (3.13)
8050 – 8089: squamous cell neoplasms	49,104 (84.57)	1759 (50.65)	31,548 (86.78)	15,797 (86.63)
8090 – 8119: basal cell neoplasms	133 (0.23)	3 (0.09)	62 (0.17)	68 (0.37)
8120 – 8139: transitional cell papillomas and carcinomas	39 (0.07)	1 (0.03)	32 (0.09)	6 (0.03)
8140 – 8389: adenomas and adenocarcinomas	2149 (3.70)	295 (8.49)	1196 (3.29)	658 (3.61)
8390 – 8429: adnexal and skin appendage neoplasms	22 (0.04)	1 (0.03)	6 (0.02)	15 (0.08)
8430 – 8439: mucoepidermoid neoplasms	2325 (4.00)	679 (19.55)	1149 (3.16)	497 (2.73)
8440 – 8499: cystic, mucinous and serous neoplasms	101 (0.17)	11 (0.32)	52 (0.14)	38 (0.21)
8500 – 8549: ductal and lobular neoplasms	416 (0.72)	50 (1.44)	213 (0.59)	153 (0.84)
8550 – 8559: acinar cell neoplasms	924 (1.59)	284 (8.18)	476 (1.31)	164 (0.90)
8560 – 8579: complex epithelial neoplasms	214 (0.37)	19 (0.55)	105 (0.29)	90 (0.49)
8930 – 8999: complex mixed and stromal neoplasms	410 (0.71)	41 (1.18)	223 (0.61)	146 (0.80)

FPHNC first primary head and neck cancer, *ICD-O-3* International Classification of Diseases for Oncology-3

Table S5 Histology recodes of FPHNC among different age groups of 2-year survivors based on histologic type ICD-O-3 [*n* (%)]

**Histology recodes**	**All the 2-year survivors (*n* = 75,209)**	**Young patients (aged 15 – 39 years) (*n* = 3847)**	**Middle-aged patients (aged 40 – 64 years) (*n* = 45,061)**	**Elderly patients (aged 65 – 89 years) (*n* = 26,301)**
8000 – 8009: unspecified neoplasms	138 (0.18)	10 (0.26)	70 (0.16)	58 (0.22)
8010 – 8049: epithelial neoplasms	2775 (3.69)	363 (9.44)	1540 (3.42)	872 (3.32)
8050 – 8089: squamous cell neoplasms	64,338 (85.55)	1987 (51.65)	39,426 (87.49)	22,925 (87.16)
8090 – 8119: basal cell neoplasms	171 (0.23)	3 (0.08)	71 (0.16)	97 (0.37)
8120 – 8139: transitional cell papillomas and carcinomas	49 (0.07)	3 (0.08)	35 (0.08)	11 (0.04)
8140 – 8389: adenomas and adenocarcinomas	2678 (3.56)	340 (8.84)	1416 (3.14)	922 (3.51)
8390 – 8429: adnexal and skin appendage neoplasms	25 (0.03)	1 (0.03)	7 (0.02)	17 (0.06)
8430 – 8439: mucoepidermoid neoplasms	2608 (3.47)	708 (18.40)	1261 (2.80)	639 (2.43)
8440 – 8499: cystic, mucinous and serous neoplasms	122 (0.16)	13 (0.34)	59 (0.13)	50 (0.19)
8500 – 8549: ductal and lobular neoplasms	510 (0.68)	53 (1.38)	256 (0.57)	201 (0.76)
8550 – 8559: acinar cell neoplasms	1029 (1.37)	296 (7.69)	527 (1.17)	206 (0.78)
8560 – 8579: complex epithelial neoplasms	268 (0.36)	23 (0.60)	131 (0.29)	114 (0.43)
8930 – 8999: complex mixed and stromal neoplasms	498 (0.66)	47 (1.22)	262 (0.58)	189 (0.72)

ICD-O-3 International Classification of Diseases for Oncology-3, *FPHNC* first primary head and neck cancer

Table S6 Collinearity diagnostics for multivariable Fine-Gray competing risk models

Independent variables	Variance inflation factor (VIF)	
	**2-year survivors (*n* = 75,209)**	**5-year survivors (*n* = 58,063)**
For analyses not stratified		
Sex	1.058	1.066
Radiotherapy	1.492	1.520
Chemotherapy	1.661	1.709
Cancer-directed surgery	1.356	1.370
FPHNC site	1.101	1.119
FPHNC histology	1.129	1.149
Age as a continuous variable	1.068	1.066
For analyses stratified by age		
Sex	1.057	1.066
Radiotherapy	1.492	1.520
Chemotherapy	1.633	1.686
Cancer-directed surgery	1.353	1.368
FPHNC site	1.083	1.102
FPHNC histology	1.102	1.117

FPHNC first primary head and neck cancer

Table S7 Collinearity diagnostics for multivariable modified Poisson regression models

Independent variables	Variance inflation factor (VIF)	
	**2-year survivors (*n* = 75,209)**	**5-year survivors (*n* = 58,063)**
For analyses not stratified		
Sex	1.028	1.032
Radiotherapy	1.404	1.428
Chemotherapy	1.428	1.445
Latency period	1.087	1.068
Age as a continuous variable	1.103	1.083
For analyses stratified by age		
Sex	1.028	1.031
Radiotherapy	1.404	1.428
Chemotherapy	1.397	1.421
Latency period	1.007	1.003

Table S8 Baseline characteristics of the 5-year survivors of FPHNC

Characteristic	All the 5-year survivors (*n* = 58,063)			Young patients (aged 15 – 39 years) (*n* = 3473)			Middle-aged patients (aged 40 – 64 years) (*n* = 36,355)			Elderly patients (aged 65 – 89 years) (*n* = 18,235)		
	Radiotherapy (*n* = 37,748)	None/Unknown (*n* = 20,315)	*P*-value[a]	Radiotherapy (*n* = 1817)	None/Unknown (*n* = 1656)	*P*-value[a]	Radiotherapy (*n* = 25,206)	None/Unknown (*n* = 11,149)	*P*-value[a]	Radiotherapy (*n* = 10,725)	None/Unknown (*n* = 7510)[a]	*P*-value[a]
Age at diagnosis [years, median (IQR)]	58 (51 − 66)	60 (51 − 69)	< 0.001	34 (28 − 37)	32 (26 − 36)	< 0.001	55 (50 − 60)	55 (49 − 60)	< 0.001	70 (67 − 75)	72 (68 − 78)	< 0.001
Sex [*n* (%)]			< 0.001			< 0.001			< 0.001			< 0.001
Male	29,333 (69.67)	12,769 (30.33)		1035 (56.96)	785 (47.40)		20,196 (80.12)	7371 (66.11)		8102 (75.54)	4613 (61.42)	
Female	8415 (52.72)	7546 (47.28)		782 (43.04)	871 (52.60)		5010 (19.88)	3778 (33.89)		2623 (24.46)	2897 (38.58)	
Race and origin [*n* (%)]			< 0.001			< 0.001			< 0.001			< 0.001
Non-Hispanic White	28,454 (64.29)	15,803 (35.71)		930 (51.18)	1011 (61.05)		19,145 (75.95)	8648 (77.57)		8379 (78.13)	6144 (81.81)	
Non-Hispanic Black	3154 (73.40)	1143 (26.60)		220 (12.11)	140 (8.45)		2208 (8.76)	710 (6.37)		726 (6.77)	293 (3.90)	
Others[b]	6140 (64.57)	3369 (35.43)		667 (36.71)	505 (30.50)		3853 (15.29)	1791 (16.06)		1620 (15.10)	1073 (14.29)	
Chemotherapy [*n* (%)]			< 0.001			< 0.001			< 0.001			< 0.001
Yes	21,824 (97.62)	533 (2.38)		1016 (55.92)	30 (1.81)		16,117 (63.94)	372 (3.34)		4691 (43.74)	131 (1.74)	
None/Unknown	15,924 (44.60)	19,782 (55.40)		801 (44.08)	1626 (98.19)		9089 (36.06)	10,777 (96.66)		6034 (56.26)	7379 (98.26)	
Cancer-directed surgery [*n* (%)]			< 0.001			< 0.001			< 0.001			< 0.001
Performed	17,085 (48.63)	18,051 (51.37)		1109 (61.03)	1538 (92.87)		11,212 (44.48)	9816 (88.04)		4764 (44.42)	6697 (89.17)	
None/Unknown	20,663 (90.13)	2264 (9.87)		708 (38.97)	118 (7.13)		13,994 (55.52)	1333 (11.96)		5961 (55.58)	813 (10.83)	
Follow-up time [*n* (%)]			< 0.001			0.59			< 0.001			< 0.001
5 – 10 years	21,300 (66.61)	10,679 (33.39)		732 (40.29)	656 (39.61)		13,219 (52.44)	5192 (46.57)		7349 (68.52)	4831 (64.33)	
10 − 15 years	12,676 (63.46)	7300 (36.54)		733 (40.34)	695 (41.97)		9054 (35.92)	4369 (39.19)		2889 (26.94)	2236 (29.77)	
> 15 years	3772 (61.76)	2336 (38.24)		352 (19.37)	305 (18.42)		2933 (11.64)	1588 (14.24)		487 (4.54)	443 (5.90)	
Follow-up time [months, median (IQR)]	112 (86 − 147)	116 (88 − 153)	< 0.001	134 (100 − 171)	135 (101 − 170)	0.96	116 (89 − 153)	124 (93 − 161)	< 0.001	99 (80 − 129)	104 (82 − 135)	< 0.001
FPHNC stage according to AJCC [*n* (%)]			< 0.001			< 0.001			< 0.001			< 0.001
0	620 (1.64)	2236 (11.01)		14 (0.77)	112 (6.76)		307 (1.22)	1204 (10.80)		299 (2.79)	920 (12.25)	
I	6394 (16.94)	9834 (48.41)		291 (16.02)	906 (54.71)		3374 (13.39)	5310 (47.63)		2729 (25.45)	3618 (48.18)	

Characteristic	All the 5-year survivors (*n* = 58,063)			Young patients (aged 15 – 39 years) (*n* = 3473)			Middle-aged patients (aged 40 – 64 years) (*n* = 36,355)			Elderly patients (aged 65 – 89 years) (*n* = 18,235)		
	**Radiotherapy (*n* = 37,748)**	**None/Unknown (*n* = 20,315)**	***P*-value[a]**	**Radiotherapy (*n* = 1817)**	**None/Unknown (*n* = 1656)**	***P*-value[a]**	**Radiotherapy (*n* = 25,206)**	**None/Unknown (*n* = 11,149)**	***P*-value[a]**	**Radiotherapy (*n* = 10,725)**	**None/Unknown (*n* = 7510)[a]**	***P*-value[a]**
II	5103 (13.52)	2594 (12.77)		353 (19.43)	253 (15.28)		3093 (12.27)	1402 (12.58)		1657 (15.45)	939 (12.50)	
III	7037 (18.64)	1227 (6.04)		457 (25.15)	102 (6.16)		4781 (18.97)	723 (6.48)		1799 (16.77)	402 (5.35)	
IV	16,700 (44.24)	1267 (6.24)		622 (34.23)	79 (4.77)		12,350 (49.00)	807 (7.24)		3728 (34.76)	381 (5.07)	
Unknown	1894 (5.02)	3157 (15.54)		80 (4.40)	204 (12.32)		1301 (5.16)	1703 (15.27)		513 (4.78)	1250 (16.64)	

[a]The continuous data were analyzed using the Mann-Whitney *U* test, while the categorical data were analyzed using the Chi-square test.

[b]Include non-Hispanic American Indian/Alaska Native, non-Hispanic Asian or Pacific Islander, non-Hispanic unknown race, and Hispanic (all races).

AJCC the American Joint Committee on Cancer, *FPHNC* first primary head and neck cancer, *IQR* interquartile range

Table S9 SIRs for solid SPMs (external comparison with the general population by radiotherapy exposure) and IRRs (internal comparison within the cohort by radiotherapy exposure) in 5-year survivors of FPHNC

Second primary malignancy site[a]	External comparison (*n* = 58,063)				Internal comparison (*n* = 58,063)	
	None/Unknown (*n* = 20,315)		Radiotherapy (*n* = 37,748)		Radiotherapy vs. None/Unknown	
	Observed	SIR (95% CI)	Observed	SIR (95% CI)	IRR[b] (95% CI)	*P*-value
All solid malignancies excluding HNC	1620	1.26 (1.20 – 1.32)	3442	1.48 (1.43 – 1.53)	1.16 (1.08 − 1.24)	< 0.001
Esophagus[c,d]	57	2.91 (2.20 – 3.77)	192	4.87 (4.21 – 5.61)	1.34 (0.92 – 1.95)	0.12
Stomach[c,e]	34	1.28 (0.88 – 1.78)	61	1.25 (0.96 – 1.61)	1.03 (0.64 – 1.69)	0.89
Small intestine	9	1.08 (0.49 – 2.05)	6	0.39 (0.14 – 0.84)	0.30 (0.06 – 1.60)	0.16
Colon and rectum[c,d]	129	0.99 (0.83 – 1.18)	264	1.16 (1.03 – 1.31)	1.05 (0.82 – 1.33)	0.72
Anus, anal canal and anorectum[f]	7	1.23 (0.50 – 2.54)	15	1.59 (0.89 – 2.63)	1.35 (0.51 – 3.57)	0.55
Liver[c,d]	40	1.26 (0.90 – 1.71)	88	1.31 (1.05 – 1.62)	1.27 (0.83 – 1.95)	0.26
Intrahepatic bile duct	10	1.82 (0.87 – 3.34)	12	1.22 (0.63 – 2.14)	0.91 (0.36 – 2.31)	0.84
Other biliary	13	1.69 (0.90 – 2.89)	11	0.84 (0.42 – 1.50)	0.41 (0.15 – 1.10)	0.08
Pancreas[c]	53	1.00 (0.75 – 1.31)	83	0.91 (0.72 – 1.13)	1.03 (0.70 – 1.52)	0.87
Other digestive organs	7	2.27 (0.91 – 4.67)	7	1.34 (0.54 – 2.77)	0.39 (0.11 – 1.38)	0.14
Lung and bronchus[c]	477	2.27 (2.07 – 2.48)	1212	3.28 (3.10 – 3.47)	1.49 (1.33 – 1.68)	< 0.001
Trachea	2	11.95 (1.45 – 43.17)	9	27.5 (12.57 – 52.20)	2.69 (0.50 – 14.53)	0.25
Bones and joints	5	2.94 (0.96 – 6.87)	21	6.91 (4.28 – 10.57)	1.83 (0.59 – 5.65)	0.30
Soft tissue including heart	16	1.62 (0.92 – 2.63)	42	2.41 (1.74 – 3.26)	1.40 (0.73 – 2.72)	0.32
Melanoma of the skin	83	0.94 (0.75 – 1.16)	147	0.92 (0.77 – 1.08)	0.91 (0.66 – 1.26)	0.56
Other non – epithelial skin	18	2.06 (1.22 – 3.25)	18	1.27 (0.75 – 2.01)	0.67 (0.30 – 1.50)	0.32

Second primary malignancy site[a]	External comparison (*n* = 58,063)				Internal comparison (*n* = 58,063)	
	None/Unknown (*n* = 20,315)		Radiotherapy (*n* = 37,748)		Radiotherapy vs. None/Unknown	
	Observed	SIR (95% CI)	Observed	SIR (95% CI)	IRR[b] (95% CI)	*P*-value
Breast[c,d,g]	119	0.89 (0.74 – 1.07)	145	0.98 (0.83 – 1.15)	1.05 (0.79 – 1.39)	0.76
Urinary bladder[c]	99	1.01 (0.82 – 1.23)	209	1.18 (1.02 – 1.35)	1.11 (0.84 – 1.46)	0.45
Kidney and renal pelvis[c]	57	0.97 (0.73 – 1.26)	101	0.87 (0.71 – 1.06)	0.78 (0.51 – 1.18)	0.24
Brain	12	0.75 (0.39 – 1.31)	29	0.99 (0.66 – 1.42)	1.13 (0.52 – 2.45)	0.76
Thyroid	22	1.06 (0.66 – 1.60)	21	0.60 (0.37 – 0.92)	0.90 (0.46 – 1.74)	0.75
Corpus and uterus[g]	27	0.93 (0.61 – 1.35)	21	0.63 (0.39 – 0.97)	1.09 (0.60 – 1.98)	0.77
Ovary[c,g]	11	0.98 (0.49 – 1.75)	5	0.42 (0.14 – 0.99)	0.36 (0.10 – 1.25)	0.11
Vulva[f,g]	7	2.11 (0.85 – 4.34)	6	1.88 (0.69 – 4.09)	1.01 (0.30 – 3.36)	0.99
Prostate[h]	280	1.01 (0.90 – 1.14)	678	1.06 (0.98 – 1.14)	1.02 (0.87 – 1.20)	0.83

[a]Only sites with more than 10 events are reported here. [b]Modified Poisson regression models, adjusted for sex, age at diagnosis of FPHNC, latency (time between FPHNC and SPM diagnoses), and chemotherapy. [c]These are organs for which a dose-response relationship with smoking has been described in the previous studies [1-7]. [d]These are organs for which a dose-response relationship with alcohol has been described in previous studies [1,8-11]. [e]These are organs possibly associated with Epstein-Barr virus (EBV) infections [12]. [f]These are organs possibly associated with human papillomavirus (HPV) infections [13]. [g]Analysis restricted only to women. [h]Analysis restricted only to men.

CI confidence interval, *FPHNC* first primary head and neck cancer, *HNC* head and neck cancer, *IRR* incidence rate ratio, *NA* not applicable, *SIR* standardized incidence ratio, *SPMs* second primary malignancies

Table S10 SIRs for solid SPMs in young 5-year survivors of FPHNC (external comparison with the general population by radiotherapy exposure)

Second primary malignancy site[a]	None/unknown		Radiotherapy	
	Observed	SIR (95% CI)	Observed	SIR (95% CI)
All solid malignancies excluding HNC	28	1.43 (0.95 – 2.06)	36	1.65 (1.16 – 2.29)
Breast[b,c,d]	9	1.54 (0.70 – 2.93)	8	1.33 (0.57 – 2.62)
Thyroid	7	3.47 (1.39 – 7.15)	4	1.98 (0.54 – 5.08)

[a]Only sites with more than 10 events are reported here. [b]These are organs for which a dose-response relationship with smoking has been described in the previous studies [1-7]. [c]These are organs for which a dose-response relationship with alcohol has been described in the previous studies [1,8-11]. [d]Analysis restricted only to women.

CI confidence interval, *FPHNC* first primary head and neck cancer, *HNC* head and neck cancer, *SIR* standardized incidence ratio, *SPMs* second primary malignancies

Table S11 SIRs for solid SPMs in middle-aged 5-year survivors of FPHNC (external comparison with the general population by radiotherapy exposure)

Second primary malignancy site[a]	None/unknown		Radiotherapy	
	**Observed**	**SIR (95% CI)**	**Observed**	**SIR (95% CI)**
All solid malignancies excluding HNC	860	1.29 (1.20 – 1.37)	2255	1.53 (1.47 – 1.59)
Esophagus[b,c]	34	3.32 (2.30 – 4.64)	134	5.30 (4.44 – 6.27)
Stomach[b,d]	14	1.14 (0.63 – 1.92)	35	1.24 (0.86 – 1.72)
Small intestine	5	1.15 (0.37 – 2.68)	5	0.51 (0.17 – 1.19)
Colon and rectum[b,c]	56	0.92 (0.69 – 1.19)	149	1.09 (0.92 – 1.28)
Anus, anal canal and anorectum[e]	4	1.20 (0.33 – 3.07)	11	1.70 (0.85 – 3.04)
Liver[b,c]	30	1.53 (1.03 – 2.18)	67	1.37 (1.07 – 1.75)
Intrahepatic bile duct	6	2.15 (0.79 – 4.67)	6	0.97 (0.36 – 2.12)
Pancreas[b]	28	1.18 (0.79 – 1.71)	52	0.99 (0.74 – 1.29)
Lung and bronchus[b]	260	2.72 (2.40 – 3.08)	781	3.74 (3.48 – 4.01)
Bones and joints	3	3.44 (0.71 – 10.06)	15	7.72 (4.32 – 12.73)
Soft tissue including heart	7	1.52 (0.61 – 3.14)	29	2.84 (1.90 – 4.07)
Melanoma of the skin	39	0.92 (0.65 – 1.26)	84	0.88 (0.70 – 1.09)
Other non – epithelial skin	6	2.14 (0.79 – 4.66)	8	1.27 (0.55 – 2.49)
Urinary bladder[b]	40	1.06 (0.76 – 1.45)	128	1.41 (1.18 – 1.68)
Kidney and renal pelvis[b]	36	1.08 (0.75 – 1.49)	72	0.92 (0.72 – 1.16)
Brain	5	0.58 (0.19 – 1.35)	17	0.88 (0.51 – 1.41)
Thyroid	13	0.97 (0.52 – 1.66)	11	0.43 (0.22 – 0.77)
Breast[b,c,f]	59	0.80 (0.61 – 1.03)	93	1.02 (0.82 – 1.25)
Corpus and uterus[f]	15	0.80 (0.45 – 1.31)	14	0.59 (0.32 – 1.00)
Vulva[e,f]	4	2.98 (0.81 – 7.62)	6	3.70 (1.36 – 8.06)
Prostate[g]	163	0.94 (0.81 – 1.1)	493	1.07 (0.98 – 1.17)

[a]Only sites with more than 10 events are reported here. [b]These are organs for which a dose-response relationship with smoking has been described in the previous studies [1-7]. [c]These are organs for which a dose-response relationship with alcohol has been described in the previous studies [1,8-11]. [d]These are organs possibly associated with Epstein-Barr virus (EBV) infections [12]. [e]These are organs possibly associated with human papillomavirus (HPV) infections [13]. [f]Analysis restricted only to women. [g]Analysis restricted only to men.

CI confidence interval, *FPHNC* first primary head and neck cancer, *HNC* head and neck cancer, *SIR* standardized incidence ratio, *SPMs* second primary malignancies

Table S12 SIRs for solid SPMs in elderly 5-year survivors of FPHNC (external comparison with the general population by radiotherapy exposure)

Second primary malignancy site[a]	None/Unknown		Radiotherapy	
	Observed	SIR (95% CI)	Observed	SIR (95% CI)
All solid malignancies excluding HNC	732	1.23 (1.14 – 1.32)	1151	1.38 (1.30 – 1.46)
Esophagus[b,c]	23	2.49 (1.58 – 3.74)	54	3.87 (2.91 – 5.05)
Stomach[b,d]	20	1.42 (0.87 – 2.20)	25	1.25 (0.81 – 1.84)
Small intestine	4	1.04 (0.28 – 2.66)	1	0.18 (0 – 1.01)
Colon and rectum[b,c]	69	1.03 (0.80 – 1.31)	113	1.29 (1.06 – 1.55)
Anus, anal canal and anorectum[e]	3	1.34 (0.28 – 3.93)	4	1.42 (0.39 – 3.64)
Liver[b,c]	10	0.83 (0.40 – 1.53)	21	1.17 (0.72 – 1.79)
Intrahepatic bile duct	4	1.50 (0.41 – 3.85)	6	1.67 (0.61 – 3.63)
Other biliary	10	2.27 (1.09 – 4.17)	8	1.35 (0.58 – 2.66)
Pancreas[b]	25	0.87 (0.56 – 1.28)	29	0.76 (0.51 – 1.09)
Lung and bronchus[b]	216	1.89 (1.65 – 2.16)	430	2.70 (2.45 – 2.97)
Soft tissue including heart	9	1.78 (0.82 – 3.39)	9	1.30 (0.59 – 2.47)
Melanoma of the skin	41	0.92 (0.66 – 1.25)	61	0.96 (0.74 – 1.24)
Other non-epithelial skin	12	2.05 (1.06 – 3.57)	9	1.16 (0.53 – 2.21)
Urinary bladder[b]	59	0.99 (0.75 – 1.27)	81	0.93 (0.74 – 1.16)
Kidney and renal pelvis[b]	20	0.82 (0.50 – 1.27)	26	0.72 (0.47 – 1.05)
Brain	7	1.02 (0.41 – 2.09)	12	1.24 (0.64 – 2.17)
Breast[b,c,f]	47	0.92 (0.68 – 1.23)	40	0.89 (0.63 – 1.21)
Corpus and uterus[f]	11	1.17 (0.58 – 2.10)	7	0.81 (0.32 – 1.66)
Prostate[g]	117	1.13 (0.94 – 1.36)	184	1.02 (0.88 – 1.18)

[a]Only sites with more than 10 events are reported here. [b]These are organs for which a dose-response relationship with smoking has been described in the previous studies [1-7]. [c]These are organs for which a dose-response relationship with alcohol has been described in the previous studies [1,8-11]. [d]These are organs possibly associated with Epstein-Barr virus (EBV) infections [12]. [e]These are organs possibly associated with human papillomavirus (HPV) infections [13]. [f]Analysis restricted only to women. [g]Analysis restricted only to men.

CI confidence interval, *FPHNC* first primary head and neck cancer, *HNC* head and neck cancer, *SIR* standardized incidence ratio, *SPMs* second primary malignancies

Table S13 Age- and sex-stratified SIRs for SPMs in survivors of FPHNC (external comparison with the general population by radiotherapy exposure)

Second primary malignancy site	None/Unknown		Radiotherapy	
	Observed	SIR (95% CI)	Observed	SIR (95% CI)
All solid malignancies excluding HNC	20,315		37,748	
Young patients (aged 15 – 39 years)				
Male	5	0.79 (0.26 – 1.84)	19	2.25 (1.35 – 3.51)
Female	23	1.73 (1.09 – 2.59)	17	1.28 (0.74 – 2.05)
Middle-aged patients (aged 40 – 64 years)				
Male	615	1.31 (1.21 – 1.42)	1856	1.51 (1.44 – 1.58)
Female	245	1.22 (1.08 – 1.39)	399	1.62 (1.46 – 1.79)
Elderly patients (aged 65 – 89 years)				
Male	507	1.23 (1.12 – 1.34)	900	1.33 (1.24 – 1.42)
Female	225	1.22 (1.07 – 1.40)	251	1.60 (1.41 – 1.81)
All hematologic malignancies	25,194		50,015	
Young patients (aged 15 – 39 years)				
Male	2	1.27 (0.15 – 4.59)	3	1.43 (0.29 – 4.17)
Female	0	0 (0 – 3.00)	3	2.52 (0.52 – 7.36)
Middle-aged patients (aged 40 – 64 years)				
Male	62	0.86 (0.66 – 1.1)	192	0.99 (0.86 – 1.15)
Female	23	0.94 (0.6 – 1.41)	27	0.87 (0.58 – 1.27)
Elderly patients (aged 65 – 89 years)				
Male	99	0.99 (0.80 – 1.20)	143	0.86 (0.73 – 1.02)
Female	39	1.00 (0.71 – 1.36)	29	0.86 (0.58 – 1.24)

CI confidence interval, *FPHNC* first primary head and neck cancer, *HNC* head and neck cancer, *SIR* standardized incidence ratio, *SPMs* second primary malignancies

Table S14 Baseline characteristics of the 2-year survivors of FPHNC

Characteristic	All the 2-year survivors (*n* = 75,209)			Aged-stratified: Young patients (aged 15 – 39 years) (*n* = 3847)			Middle-aged patients (aged 40 – 64 years) (*n* = 45,061)			Elderly patients (aged 65 – 89 years) (*n* = 26,301)		
	Radiotherapy (*n* = 50,015)	None/Unknown (*n* = 25,194)	*P*-value[a]	Radiotherapy (*n* = 2078)	None/Unknown (*n* = 1769)	*P*-value[a]	Radiotherapy (*n* = 31,898)	None/Unknown (*n* = 13,163)	*P*-value[a]	Radiotherapy (*n* = 16,039)	None/Unknown (*n* = 10,262)	*P*-value[a]
Age at diagnosis [years, median (IQR)]	59 (52 – 67)	61 (52 – 71)	< 0.001	34 (28 – 37)	32 (26 – 36)	< 0.001	55 (50 – 60)	55 (50 – 60)	< 0.001	71 (67 – 76)	73 (68 – 79)	< 0.001
Sex [*n* (%)]			< 0.001			< 0.001			< 0.001			< 0.001
Male	38,924 (70.75)	16,092 (29.25)		1205 (57.99)	844 (47.71)		25,608 (80.28)	8833 (67.10)		12,111 (75.51)	6415 (62.51)	
Female	11,091 (54.92)	9102 (45.08)		873 (42.01)	925 (52.29)		6290 (19.72)	4330 (32.90)		3928 (24.49)	3847 (37.49)	
Race and origin [*n* (%)]			< 0.001			< 0.001			< 0.001			< 0.001
Non-Hispanic White	37,521 (65.64)	19,640 (34.36)		1042 (50.14)	1066 (60.26)		23,938 (75.05)	10,174 (77.29)		12,541 (78.19)	8400 (81.86)	
Non-Hispanic Black	4575 (75.38)	1494 (24.62)		255 (12.27)	146 (8.25)		3121 (9.78)	904 (6.87)		1199 (7.48)	444 (4.33)	
Others[b]	7919 (66.11)	4060 (33.89)		781 (37.58)	557 (31.49)		4839 (15.17)	2085 (15.84)		2299 (14.33)	1418 (13.82)	
Chemotherapy [*n* (%)]			< 0.001			< 0.001			< 0.001			< 0.001
Yes	28,967 (97.38)	778 (2.62)		1199 (57.70)	37 (2.09)		20,630 (64.67)	531 (4.03)		7138 (44.50)	210 (2.05)	
None/unknown	21,048 (46.30)	24,416 (53.70)		879 (42.30)	1732 (97.91)		11,268 (35.33)	12,632 (95.97)		8901 (55.50)	10,052 (97.95)	
Cancer-directed surgery [*n* (%)]			< 0.001			< 0.001			< 0.001			< 0.001
Performed	22,004 (49.95)	22,045 (50.05)		1261 (60.68)	1638 (92.59)		13,843 (43.40)	11,382 (86.47)		6900 (43.02)	9025 (87.95)	
None/unknown	28,011 (89.89)	3149 (10.11)		817 (39.32)	131 (7.41)		18,055 (56.60)	1781 (13.53)		9139 (56.98)	1237 (12.05)	
Follow-up time [*n* (%)]			< 0.001			< 0.001			< 0.001			< 0.001
2 – 10 years	32,071 (68.54)	14,722 (31.46)		980 (47.16)	750 (42.40)		18,894 (59.23)	6707 (50.95)		12,197 (76.05)	7265 (70.80)	
10 – 15 years	13,737 (63.53)	7885 (36.47)		736 (35.42)	704 (39.80)		9732 (30.51)	4691 (35.64)		3269 (20.38)	2490 (24.26)	
> 15 years	4207 (61.92)	2587 (38.08)		362 (17.42)	315 (17.81)		3272 (10.26)	1765 (13.41)		573 (3.57)	507 (4.94)	
Follow-up time [months, median (IQR)]	99 (70 – 139)	108 (77 – 148)	< 0.001	124 (87 – 167)	131 (96 – 169)	< 0.001	106 (75 – 146)	118 (84 – 158)	< 0.001	85 (54 – 118)	93 (66 – 126)	< 0.001
FPHNC stage according to AJCC [*n* (%)]			< 0.001			< 0.001			< 0.001			< 0.001

Characteristic	All the 2-year survivors (*n* = 75,209)			Aged-stratified: Young patients (aged 15 – 39 years) (*n* = 3847)			Middle-aged patients (aged 40 – 64 years) (*n* = 45,061)			Elderly patients (aged 65 – 89 years) (*n* = 26,301)		
	**Radiotherapy (*n* = 50,015)**	**None/Unknown (*n* = 25,194)**	***P*-value[a]**	**Radiotherapy (*n* = 2078)**	**None/Unknown (*n* = 1769)**	***P*-value[a]**	**Radiotherapy (*n* = 31,898)**	**None/Unknown (*n* = 13,163)**	***P*-value[a]**	**Radiotherapy (*n* = 16,039)**	**None/Unknown (*n* = 10,262)**	***P*-value[a]**
0	760 (1.52)	2746 (10.90)		14 (0.67)	118 (6.67)		348 (1.09)	1396 (10.61)		398 (2.48)	1232 (12.01)	
I	8136 (16.27)	11,793 (46.81)		310 (14.92)	951 (53.76)		4036 (12.65)	6076 (46.16)		3790 (23.63)	4766 (46.44)	
II	6731 (13.46)	3283 (13.03)		388 (18.67)	275 (15.55)		3845 (12.05)	1679 (12.76)		2498 (15.57)	1329 (12.95)	
III	9382 (18.76)	1643 (6.52)		516 (24.83)	116 (6.56)		6049 (18.96)	893 (6.78)		2817 (17.56)	634 (6.18)	
IV	22,517 (45.02)	1828 (7.26)		755 (36.33)	88 (4.97)		16,006 (50.18)	1,126 (8.55)		5756 (35.89)	614 (5.98)	
Unknown	2489 (4.98)	3901 (15.48)		95 (4.57)	221 (12.49)		1614 (5.06)	1993 (15.14)		780 (4.86)	1687 (16.44)	

[a]The continuous data were analyzed using the Mann-Whitney *U* test, while the categorical data were analyzed using the Chi-square test. [b]Include non-Hispanic American Indian/Alaska Native, non-Hispanic Asian or Pacific Islander, non-Hispanic unknown race, and Hispanic (all races).

AJCC the American Joint Committee on Cancer, *FPHNC* first primary head and neck cancer, *IQR* interquartile range

Table S15 SIRs for hematologic SPMs (external comparison with the general population by radiotherapy exposure) and IRRs (internal comparison within the cohort by radiotherapy exposure) in 2-year survivors of FPHNC

Second primary malignancy site	External comparison (*n* = 75,209)				Internal comparison (*n* = 75,209)	
	None/Unknown (*n* = 25,194)		Radiotherapy (*n* = 50,015)		Radiotherapy vs. None/Unknown	
	Observed	SIR (95% CI)	Observed	SIR (95% CI)	IRR[a] (95% CI)	*P*-value
All hematologic malignancies	225	0.94 (0.82 – 1.07)	397	0.93 (0.84 – 1.03)	0.88 (0.72 – 1.06)	0.17
Hodgkin lymphoma	7	1.22 (0.49 – 2.51)	4	0.37 (0.10 – 0.95)	0.41 (0.11 – 1.48)	0.17
Non-Hodgkin lymphoma	121	1.08 (0.90 – 1.30)	191	0.97 (0.84 – 1.12)	0.86 (0.66 – 1.11)	0.24
Myeloma	36	0.84 (0.59 – 1.17)	60	0.75 (0.57 – 0.97)	0.95 (0.59 – 1.53)	0.82
Leukemia	61	0.77 (0.59 – 0.99)	142	1.02 (0.86 – 1.20)	0.93 (0.64 – 1.35)	0.69
Lymphocytic leukemia	23	0.59 (0.37 – 0.88)	45	0.64 (0.46 – 0.85)	1.00 (0.55 – 1.82)	0.99
Non-lymphocytic leukemia	38	0.95 (0.67 – 1.30)	97	1.41 (1.14 – 1.72)	0.89 (0.55 – 1.43)	0.63

[a]Modified Poisson regression models, adjusted for sex, age at diagnosis of FPHNC, latency (time between FPHNC and SPM diagnoses), and chemotherapy.

CI confidence interval, *FPHNC* first primary head and neck cancer, *IRR* incidence rate ratio, *SIR* standardized incidence ratio, *SPMs* second primary malignancies

Table S16 Age-stratified SIRs for hematologic SPMs, in 2-year survivors of FPHNC: external comparison with the general population by radiotherapy exposure

Second primary malignancy site	Young patients (aged 15 – 39 years) (*n* = 3847)				Middle-aged patients (aged 40 – 64 years) (*n* = 45,061)				Elderly patients (aged 65 – 89 years) (*n* = 26,301)			
	None/unknown (*n* = 1769)		Radiotherapy (*n* = 2078)		None/unknown (*n* = 13,163)		Radiotherapy (*n* = 31,898)		None/unknown (*n* = 10,262)		Radiotherapy (*n* = 16,039)	
	Observed	SIR (95% CI)	Observed	SIR (95% CI)	Observed	SIR (95% CI)	Observed	SIR (95% CI)	Observed	SIR (95% CI)	Observed	SIR (95% CI)
All hematologic malignancies	2	0.71 (0.09 – 2.58)	6	1.82 (0.67 – 3.97)	85	0.88 (0.70 – 1.09)	219	0.98 (0.85 – 1.12)	138	0.99 (0.83 – 1.17)	172	0.86 (0.74 – 1.00)
Hodgkin lymphoma	0	0 (0 – 8.59)	0	0 (0 – 8.1)	5	1.74 (0.56 – 4.06)	0	0 (0 – 0.55)	2	0.82 (0.10 – 2.95)	4	1.11 (0.30 – 2.84)
Non-Hodgkin lymphoma	1	0.8 (0.02 – 4.46)	6	4.01 (1.47 – 8.74)	45	0.98 (0.72 – 1.32)	97	0.93 (0.76 – 1.14)	75	1.16 (0.91 – 1.45)	88	0.97 (0.78 – 1.20)
Myeloma	0	0 (0 – 13.31)	0	0 (0 – 10.04)	17	0.95 (0.55 – 1.52)	39	0.92 (0.65 – 1.25)	19	0.77 (0.47 – 1.21)	21	0.57 (0.35 – 0.87)
Leukemia	1	1.18 (0.03 – 6.59)	0	0 (0 – 3.78)	18	0.59 (0.35 – 0.94)	83	1.18 (0.94 – 1.46)	42	0.88 (0.63 – 1.18)	59	0.87 (0.66 – 1.12)
Lymphocytic leukemia	0	0 (0 – 11.99)	0	0 (0 – 10.41)	6	0.38 (0.14 – 0.82)	29	0.77 (0.52 – 1.11)	17	0.74 (0.43 – 1.19)	16	0.49 (0.28 – 0.79)
Non-lymphocytic leukemia	1	1.86 (0.05 – 10.36)	0	0 (0 – 5.93)	12	0.83 (0.43 – 1.45)	54	1.63 (1.23 – 2.13)	25	1.00 (0.65 – 1.47)	43	1.22 (0.89 – 1.65)

CI confidence interval, *FPHNC* first primary head and neck cancer, *SIR* standardized incidence ratio, *SPMs* second primary malignancies

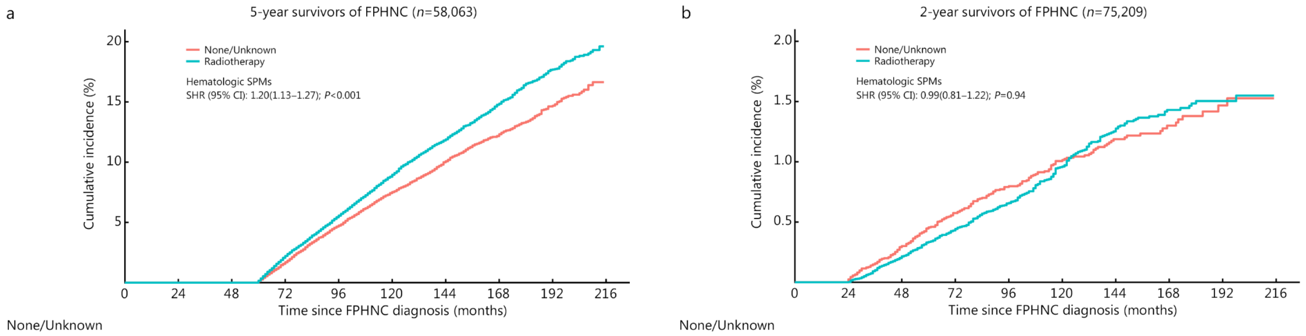

a

	0	24	48	72	96	120	144	168	192	216
None/Unknown										
At risk	20,315	20,315	20,315	19,036	13,833	9592	6200	3528	1480	0
Events	0	0	0	336	850	1196	1412	1536	1601	1620
Radiotherapy										
At risk	37,748	37,748	37,748	34,965	24,542	16,381	10,252	5615	2346	0
Events	0	0	0	813	1836	2574	3013	3279	3414	3442

b

	0	24	48	72	96	120	144	168	192	216
None/Unknown										
At risk	25,194	25,131	22,022	19,432	14,097	9757	6285	3575	1499	0
Events	0	6	71	170	850	198	213	219	224	225
Radiotherapy										
At risk	50,015	49,806	41,119	35,307	24,737	16,498	10,319	5649	2358	0
Events	0	1	97	188	258	327	377	392	396	397

Fig. S1 Associations between radiotherapy and cumulative incidence of SPMs. Multivariable Fine-Gray models adjusted for sex, age at diagnosis of FPHNC, FPHNC site, FPHNC histology, cancer-directed surgery, and chemotherapy. **a** Among 5-year survivors of non-metastatic primary head and neck cancer, radiotherapy was associated with a higher risk of developing solid SPMs, as determined by multivariable Fine-Gray competing risk models (SHR = 1.20, 95% CI 1.13 − 1.27; $P < 0.001$). **b** Multivariable Fine-Gray competing risk models showed no significant association between radiotherapy and the cumulative incidence of hematologic SPMs among 2-year survivors of non-metastatic primary head and neck cancer (SHR = 0.99, 95% CI 0.81 − 1.22; $P = 0.94$). CI confidence interval, FPHNC first primary head and neck cancer, SHR subdistribution hazard ratio, SPMs second primary malignancies

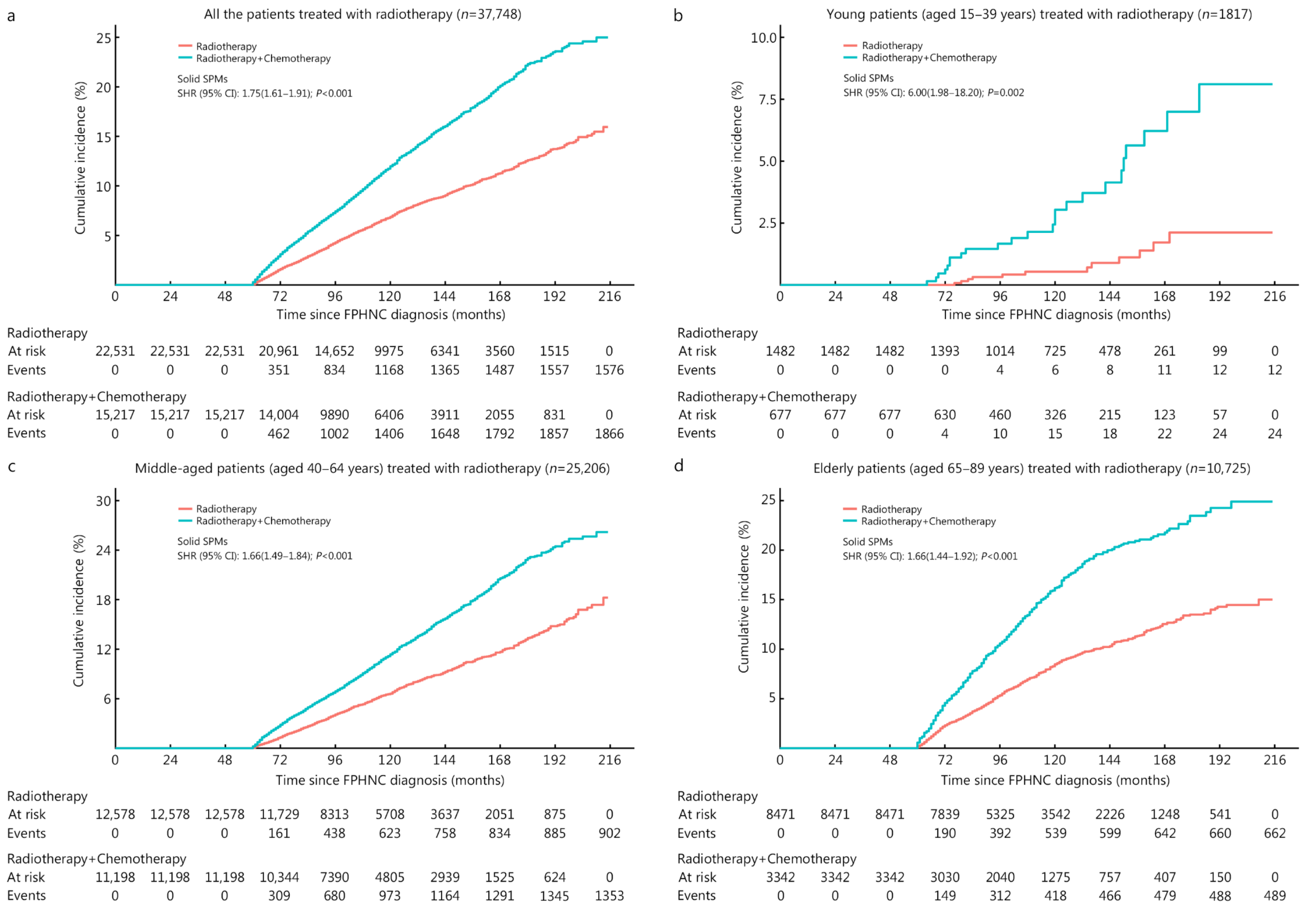

Fig. S2 Associations between chemotherapy and cumulative incidence of solid SPMs among 5-year survivors treated with radiotherapy. **a** Patients treated with radiotherapy and chemotherapy had a higher risk of developing solid SPMs, compared to patients who received radiotherapy alone (SHR = 1.75, 95% CI 1.61 − 1.91; P < 0.001). Multivariable Fine-Gray models adjusted for sex, age at diagnosis of FPHNC, FPHNC site, FPHNC histology, and cancer-directed surgery. **b** Among young patients treated with radiotherapy, chemotherapy was associated with a higher risk of developing solid SPMs (SHR = 6.00, 95% CI 1.98 − 18.20; P = 0.002). **c** Among middle-aged patients treated with radiotherapy, chemotherapy was associated with a higher risk of developing solid SPMs (SHR = 1.66, 95% CI 1.49 − 1.84; P < 0.001). **d** Among elderly patients treated with radiotherapy, chemotherapy was associated with a higher risk of developing solid SPMs (SHR = 1.66, 95% CI 1.44 − 1.92; P < 0.001). Multivariable Fine-Gray models adjusted for sex, FPHNC site, FPHNC histology, and cancer-directed surgery (age excluded due to stratification) (**b-d**). CI confidence interval, FPHNC first primary head and neck cancer, SHR subdistribution hazard ratio, SPMs second primary malignancies

Second primary malignancy site	No. of cases		IRR[a] (95% CI)	*P*-value
	Radiotherapy	None/Unknown		
**Solid malignancies: 5-year latency**				
All solid malignancies excluding HNC[b]	3442	1620	1.16(1.08–1.24)	<0.001
Lung and bronchus	1212	477	1.49(1.33–1.68)	<0.001
Prostate[c]	678	280	1.02(0.87–1.20)	0.83
Colon and rectum	264	129	1.05(0.82–1.33)	0.72
Urinary bladder	209	99	1.11(0.84–1.46)	0.45
Esophagus	192	57	1.34(0.92–1.95)	0.12
Melanoma of the skin	147	83	0.91(0.66–1.26)	0.56
Breast[d]	145	119	1.05(0.79–1.39)	0.76
Kidney and renal pelvis	101	57	0.78(0.51–1.18)	0.24
Liver	88	40	1.27(0.83–1.95)	0.26
Pancreas	83	53	1.03(0.70–1.52)	0.87
Stomach	61	34	1.03(0.64–1.69)	0.89
Soft tissue including heart	42	16	1.40(0.73–2.72)	0.32
Brain	29	12	1.13(0.52–2.45)	0.76
Thyroid	21	22	0.90(0.46–1.74)	0.75
Bones and joints	21	5	1.83(0.59–5.65)	0.30
Corpus and uterus[d]	21	27	1.09(0.60–1.98)	0.77
Other non-epithelial skin	18	18	0.67(0.30–1.50)	0.32
Anus, anal canal and anorectum	15	7	1.35(0.51–3.57)	0.55
Intrahepatic bile duct	12	10	0.91(0.36–2.31)	0.84
Other biliary	11	13	0.41(0.15–1.10)	0.08
**Hematologic malignancies: 2-year latency**				
All hematologic malignancies	397	225	0.88(0.72–1.06)	0.17
Non-Hodgkin lymphoma	191	121	0.86(0.66–1.11)	0.24
Myeloma	60	36	0.95(0.59–1.53)	0.82
Hodgkin lymphoma	4	7	0.41(0.11–1.48)	0.17
Leukemia	142	61	0.93(0.64–1.35)	0.69
Lymphocityc leukemia	45	23	1.00(0.55–1.82)	0.99
Nonlymphocytic leukemia	97	38	0.89(0.55–1.43)	0.63

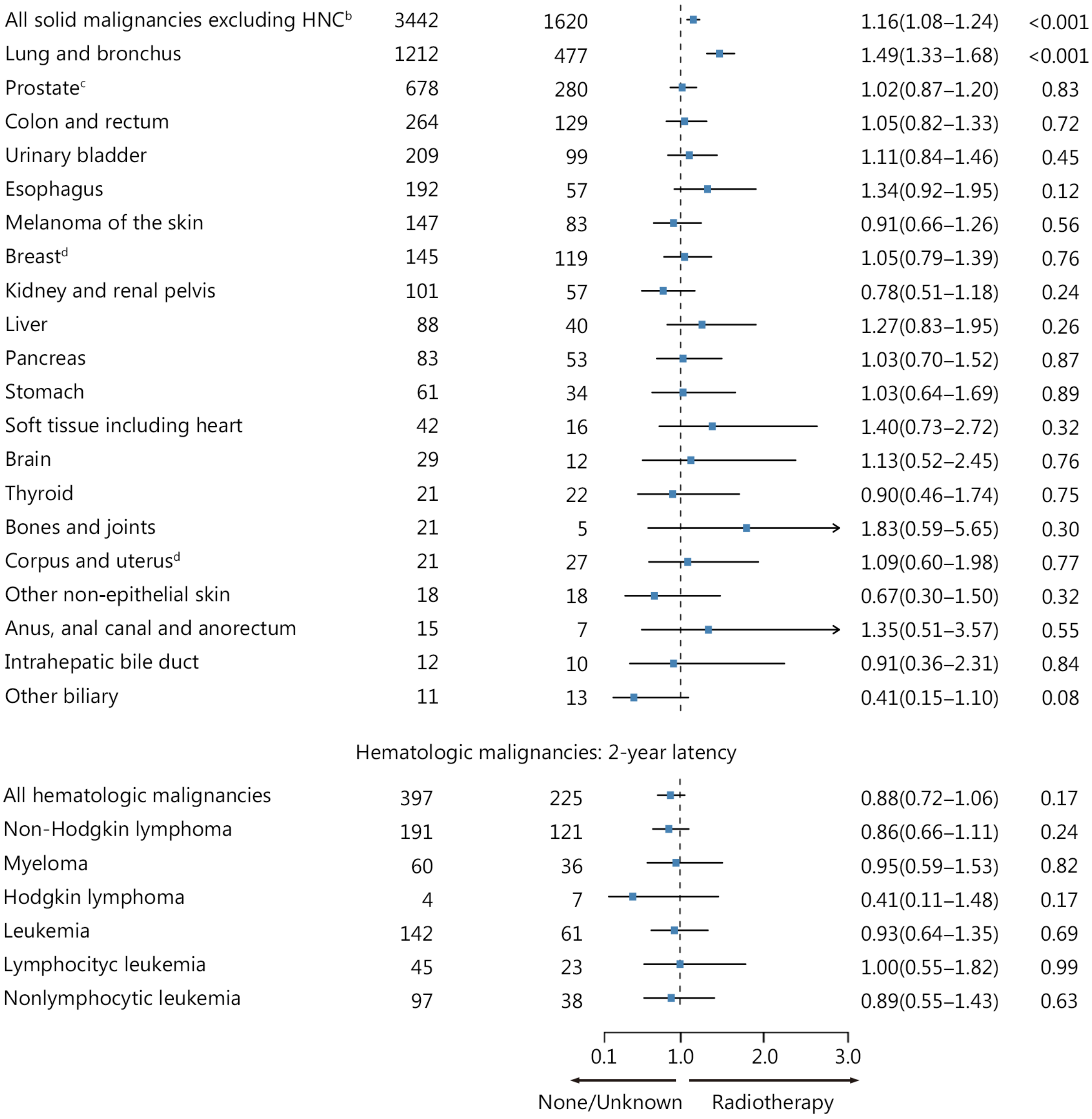

Fig. S3 IRRs and 95% CIs for solid and hematologic SPMs associated with radiotherapy in FPHNC. [a]Modified Poisson regression models, adjusted for sex, age at diagnosis of FPHNC, latency (time between FPHNC and SPM diagnoses), and chemotherapy. [b]The category "all solid malignancies" includes the ones listed in this figure as well as those malignancy sites with < 10 events for patients who received radiotherapy (**Additional file 1: Table S9** for details). [c]Analysis restricted only to men. [d]Analysis restricted only to women. CI confidence interval, FPHNC first primary head and neck cancer, HNC head and neck cancer, IRR incidence rate ratio, SPMs second primary malignancies

Second primary malignancy site	No. of cases: Radiotherapy	No. of cases: None/Unknown	IRR[a] (95% CI)	*P*-value
**Solid malignancies: 5-year latency**				
Young patients (aged 15–39 years)				
All solid malignancies excluding HNC[b]	36	28	0.97(0.53–1.76)	0.92
Breast	8	9	0.97(0.32–2.90)	0.96
Thyroid	4	7	0.89(0.24–3.33)	0.87
Middle-aged patients (aged 40–64 years)				
All solid malignancies excluding HNC[b]	2255	860	1.21(1.11–1.32)	<0.001
Lung and bronchus	781	260	1.51(1.28–1.76)	<0.001
Prostate[c]	493	163	1.16(0.95–1.43)	0.14
Colon and rectum	149	56	0.96(0.66–1.38)	0.82
Esophagus	134	34	1.41(0.88–2.26)	0.16
Urinary bladder	128	40	1.25(0.82–1.90)	0.30
Breast[d]	95	62	1.16(0.80–1.69)	0.44
Melanoma of the skin	84	39	0.80(0.50–1.29)	0.36
Kidney and renal pelvis	72	36	0.78(0.45–1.33)	0.35
Liver	67	30	1.26(0.78–2.01)	0.35
Pancreas	52	28	1.09(0.64–1.83)	0.76
Stomach	35	14	1.26(0.60–2.65)	0.55
Soft tissue including heart	29	7	1.79(0.71–4.51)	0.22
Brain	17	5	1.66(0.55–4.98)	0.37
Bones and joints	15	3	1.66(0.41–6.73)	0.48
Corpus and uterus[d]	14	15	1.20(0.56–2.58)	0.64
Thyroid	11	13	0.52(0.20–1.34)	0.17
Anus, anal canal and anorectum	11	4	1.66(0.48–5.72)	0.43
Other non-epithelial skin	8	6	0.61(0.13–2.98)	0.54
Intrahepatic bile duct	6	6	0.89(0.25–3.13)	0.85
Elderly patients (aged 65–89 years)				
All solid malignancies excluding HNC[b]	1151	732	1.06(0.96–1.17)	0.27
Lung and bronchus	430	216	1.42(1.19–1.70)	<0.001
Prostate[c]	184	117	0.76(0.58–0.99)	0.04
Colon and rectum	113	69	1.14(0.82–1.58)	0.45
Urinary bladder	81	59	0.99(0.68–1.42)	0.93
Melanoma of the skin	61	41	1.01(0.64–1.60)	0.96
Esophagus	54	23	1.14(0.62–2.09)	0.68
Breast[d]	42	48	0.96(0.59–1.56)	0.88
Pancreas	29	25	0.92(0.51–1.67)	0.79
Kidney and renal pelvis	26	20	0.68(0.35–1.34)	0.27
Stomach	25	20	0.79(0.41–1.51)	0.47
Liver	21	10	1.15(0.45–2.91)	0.78
Brain	12	7	0.72(0.24–2.17)	0.56
Soft tissue including heart	9	9	0.85(0.31–2.34)	0.76
Other non-epithelial skin	9	12	0.67(0.26–1.72)	0.41
Other biliary	8	10	0.48(0.16–1.47)	0.20
Corpus and uterus[d]	7	11	0.96(0.36–2.54)	0.93
**Hematologic malignancies: 2-year latency**				
Middle-aged patients (aged 40–64 years)				
All hematologic malignancies	219	85	0.99(0.73–1.33)	0.94
Non-Hodgkin lymphoma	97	45	0.86(0.57–1.30)	0.48
Myeloma	39	17	1.02(0.51–2.03)	0.96
Leukemia	83	18	1.54(0.83–2.88)	0.17
Lymphocityc leukemia	29	6	2.04(0.74–5.61)	0.17
Nonlymphocytic leukemia	54	12	1.30(0.59–2.87)	0.52
Elderly patients (aged 65–89 years)				
All hematologic malignancies	172	138	0.77(0.60–0.99)	0.045
Non-Hodgkin lymphoma	88	75	0.81(0.58–1.13)	0.21
Myeloma	21	19	0.85(0.44–1.65)	0.63
Leukemia	59	42	0.66(0.40–1.07)	0.09
Lymphocityc leukemia	16	17	0.60(0.26–1.37)	0.22
Nonlymphocytic leukemia	43	25	0.70(0.38–1.28)	0.25

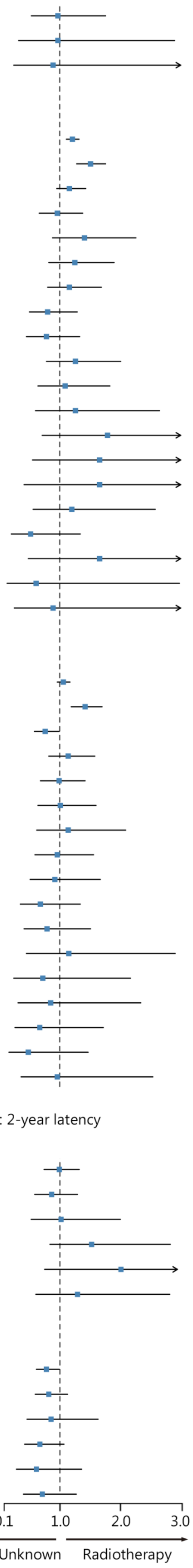

Fig. S4 Age-stratified IRRs and 95% CIs for SPMs associated with radiotherapy in FPHNC. [a]Modified Poisson regression models, adjusted for sex, latency (time between FPHNC and SPM diagnoses), and chemotherapy. [b]The category "all solid malignancies" include the ones listed in this figure as well as those malignancy sites with < 10 events. [c]Analysis restricted only to men. [d]Analysis restricted only to women. CI confidence interval, HNC head and neck cancer, IRR incidence rate ratio, FPHNC first primary head and neck cancer, SPMs second primary malignancies

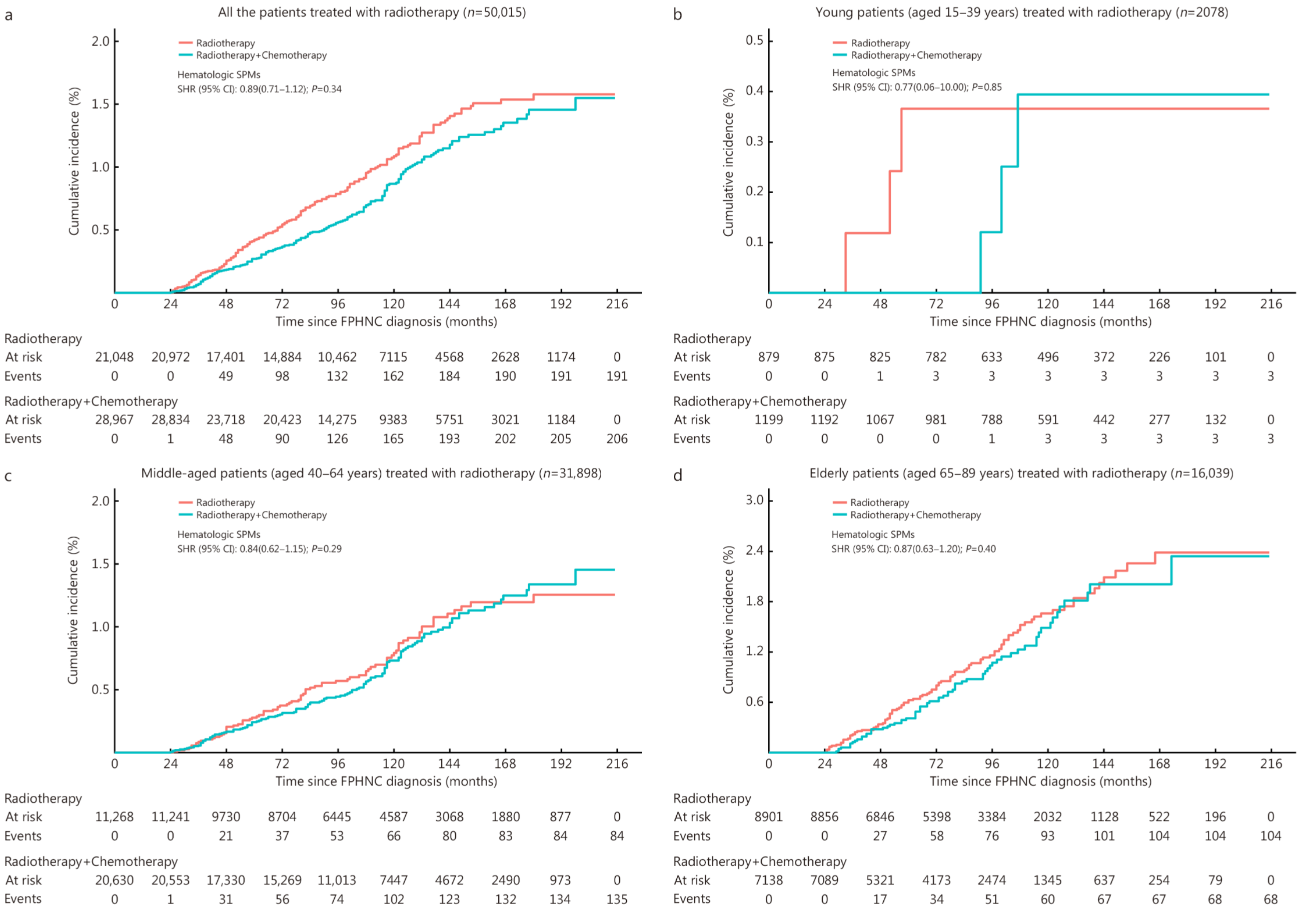

Fig. S5 Associations between chemotherapy and cumulative incidence of hematologic SPMs among 2-year survivors treated with radiotherapy. **a** No significant difference in the risk of developing hematologic SPMs was observed between patients who underwent radiotherapy alone and those treated with a combination of radiotherapy and chemotherapy. Multivariable Fine-Gray models adjusted for sex, age at diagnosis of FPHNC, FPHNC site, FPHNC histology, and cancer-directed surgery. **b** No significant difference in the risk of developing hematologic SPMs was observed between patients who underwent radiotherapy alone and those treated with a combination of radiotherapy and chemotherapy among young patients. **c** No significant difference in the risk of developing hematologic SPMs was observed between patients who underwent radiotherapy alone and those treated with a combination of radiotherapy and chemotherapy among middle-aged patients. **d** No significant difference in the risk of developing hematologic SPMs was observed between patients who underwent radiotherapy alone and those treated with a combination of radiotherapy and chemotherapy among elderly patients. Multivariable Fine-Gray models adjusted for sex, FPHNC site, FPHNC histology, and cancer-directed surgery (age excluded due to stratification) (**b-d**). CI confidence interval, FPHNC first primary head and neck cancer, SHR subdistribution hazard ratio, SPMs second primary malignancies

References

1. IARC. A Review of Human Carcinogens. Part E: Personal Habits and Indoor Combustions. Lyon, France: IARC; 2012.
2. Ai F, Zhao J, Yang W, Wan X. Dose-response relationship between active smoking and lung cancer mortality/prevalence in the Chinese population: a meta-analysis. BMC Public Health. 2023;23(1):747.
3. Nam DJ, Oh CM, Ha E, Kim MH, Yang EH, Lee HC, et al. The association of pancreatic cancer incidence with smoking status and smoking amount in Korean men. Epidemiol Health. 2022;44:e2022040.
4. Rota M, Possenti I, Valsassina V, Santucci C, Bagnardi V, Corrao G, et al. Dose-response association between cigarette smoking and gastric cancer risk: a systematic review and meta-analysis. Gastric Cancer. 2024;27(2):197-209.
5. Akter S, Islam Z, Mizoue T, Sawada N, Ihira H, Tsugane S, et al. Smoking and colorectal cancer: a pooled analysis of 10 population-based cohort studies in Japan. Int J Cancer. 2021;148(3):654-64.
6. Masaoka H, Matsuo K, Oze I, Kimura T, Tamakoshi A, Sugawara Y, et al. Cigarette smoking, smoking cessation, and bladder cancer risk: a pooled analysis of 10 cohort studies in Japan. J Epidemiol. 2023;33(11):582-8.
7. Scala M, Bosetti C, Bagnardi V, Possenti I, Specchia C, Gallus S, et al. Dose-response relationships between cigarette smoking and breast cancer risk: a systematic review and meta-analysis. J Epidemiol. 2023;33(12):640-8.
8. Im PK, Millwood IY, Kartsonaki C, Guo Y, Chen Y, Turnbull I, et al. Alcohol drinking and risks of liver cancer and non-neoplastic chronic liver diseases in China: a 10-year prospective study of 0.5 million adults. BMC Med. 2021;19(1):216.
9. Zhou X, Yu L, Wang L, Xiao J, Sun J, Zhou Y, et al. Alcohol consumption, blood DNA methylation and breast cancer: a Mendelian randomisation study. Eur J Epidemiol. 2022;37(7):701-12.
10. Im PK, Millwood IY, Kartsonaki C, Chen Y, Guo Y, Du H, et al. Alcohol drinking and risks of total and site-specific cancers in China: a 10-year prospective study of 0.5 million adults. Int J Cancer. 2021;149(3):522-34.

11. Jin EH, Han K, Shin CM, Lee DH, Kang SJ, Lim JH, et al. Sex and tumor-site differences in the association of alcohol intake with the risk of early-onset colorectal cancer. J Clin Oncol. 2023;41(22):3816-25.
12. Hirabayashi M, Georges D, Clifford GM, de Martel C. Estimating the global burden of Epstein-Barr virus-associated gastric cancer: a systematic review and meta-analysis. Clin Gastroenterol Hepatol. 2023;21(4):922-30.e21.
13. Malagón T, Franco EL, Tejada R, Vaccarella S. Epidemiology of HPV-associated cancers past, present and future: towards prevention and elimination. Nat Rev Clin Oncol. 2024;21(7):522-38.
